# A novel protein encoded by circUBE2G1 suppresses glycolysis in gastric cancer through binding to ENO1

**DOI:** 10.1038/s41420-025-02644-0

**Published:** 2025-07-29

**Authors:** Lu Lu, Guoqing Guo, Jiahao Guo, Hanyang Li, Kexin Chen, Yuli Chen, Qiuhui Li, Qiunuo Li, Yuhao Diao, Ming Sun, Hao Wu, Xianghua Liu

**Affiliations:** 1 Department of Biochemistry and Molecular Biology, School of Basic Medical Sciences, Nanjing Medical University, Nanjing, China; 2 Suzhou Cancer Center Core Laboratory, The Affiliated Suzhou Hospital of Nanjing Medical University, Suzhou Municipal Hospital, Gusu School, Nanjing Medical University, Suzhou, China; 3 The First Clinical Medical College, Nanjing Medical University, Nanjing, China; 4 Department of Oncology, The First Affiliated Hospital of Nanjing Medical University, Nanjing, Jiangsu Province China; 5 Gastric Cancer Center, The First Affiliated Hospital of Nanjing Medical University, Nanjing, Jiangsu Province PR China; 6 Institute for Gastric Cancer Research, Nanjing Medical University, Nanjing, Jiangsu China; 7 Department of Oncology, The Affiliated Jiangning Hospital of Nanjing Medical University, Nanjing, China

**Keywords:** Gastric cancer, Glycobiology

## Abstract

Gastric cancer (GC), a malignant neoplasm originating in the stomach epithelium, is characterized by substantial global incidence and mortality rates, posing a substantial threat to public health systems worldwide. The present study was designed to identify and validate a previously unannotated protein encoded by circular RNA (circRNA), with the principal objective of elucidating its functional significance and mechanistic basis in gastric carcinogenesis.CircUBE2G1 (hsa_circ_003239) was identified as a translationally active circRNA exhibiting significant downregulation in gastric cancer. The novel protein product derived from circUBE2G1 translation, designated circUBE2G1-99aa, was confirmed through co-immunoprecipitation coupled with tandem mass spectrometry (LC-MS/MS), representing the first documentation of its existence in human malignancies.CircUBE2G1-99aa exhibited marked downregulation in gastric cancer (GC), with its diminished expression levels demonstrating significant correlations with larger primary tumor size, lymph node metastasis, and advanced TNM stages. Functionally, circUBE2G1 exerted tumor-suppressive effects via its encoded protein circUBE2G1-99aa, not the full-length RNA, by inhibiting GC cell proliferation in vitro and in vivo. Mechanistically, circUBE2G1-99aa directly bound ENO1 and suppressed its glycolytic activity, thereby reducing glycolysis in GC cells. These findings delineate the functional and mechanistic landscape of circUBE2G1-99aa in gastric cancer, proposing its dual utility as both a prognostic biomarker and therapeutic target in clinical oncology.

## Background

Gastric cancer (GC) ranks fifth worldwide in terms of incidence and mortality. The pathogenesis of GC is driven by pathobiological determinants including tumor immune escape mechanisms, dynamic remodeling of the tumor microenvironment, metabolic reprogramming, and dysregulation of oncogenic signaling cascades, which have been shown to collectively orchestrate malignant cellular proliferation, invasive potential, and metastatic dissemination, ultimately manifesting as compromised therapeutic efficacy and diminished survival outcomes in clinical settings [1, 2]. Therefore, exploring how gastric cancer develops at the molecular level is key to advancing research and treatment. This understanding helps identify promising treatment targets and biomarkers for diagnosis and prognosis [3–5].

Circular RNAs (circRNAs) are defined as a novel class of non-coding RNAs with covalently closed structures [6, 7]. High-throughput sequencing and bioinformatics have identified numerous circRNAs that encode microproteins. These microproteins significantly influence tumor progression, metastasis, apoptosis, and immune evasion, presenting potential biomarkers and therapeutic targets in cancer [8, 9]. For instance, CircMAPK14-175aa inhibits colorectal cancer progression by modulating the MAPK pathway [10]. Additionally, EZH2-92aa, translated from CircEZH2, has been found to contribute to immune evasion in glioblastoma [11]. In GC, CircAXIN1-encoded AXIN1-295aa activates Wnt signaling to promote oncogenic traits, while CircGSPT1-encoded GSPT1-238aa inhibits PI3K/AKT/mTOR signaling and autophagy [12, 13]. However, there is a lack of reports detailing their functions and mechanisms of action in the context of tumor metabolic regulation, particularly regarding their role in the rewiring of energy metabolism in GC.

In this study, we analyzed circRNA microarray data alongside translational genomics data from GC cells and tissues, identifying a circRNA, circUBE2G1, with low expression and coding potential in GC. Analytical findings indicate an association between reduced expression of circUBE2G1 and its derivative microprotein, circUBE2G1-99aa, with advanced disease characteristics in GC. Experimental evidence suggests that circUBE2G1-99aa inhibits GC cell proliferation, potentially through binding to the ENO1 enzyme and modulating the ENO1/PI3K/AKT pathway, thereby suppressing glycolysis and altering tumor metabolism. Our findings provide novel insights into the role of circRNA-encoded microproteins in GC progression and metabolic regulation, advancing our understanding of oncogenesis and informing therapeutic strategies.

## Results

### Identification of circUBE2G1 and the clinical features of circUBE2G1

**Fig. 1:**
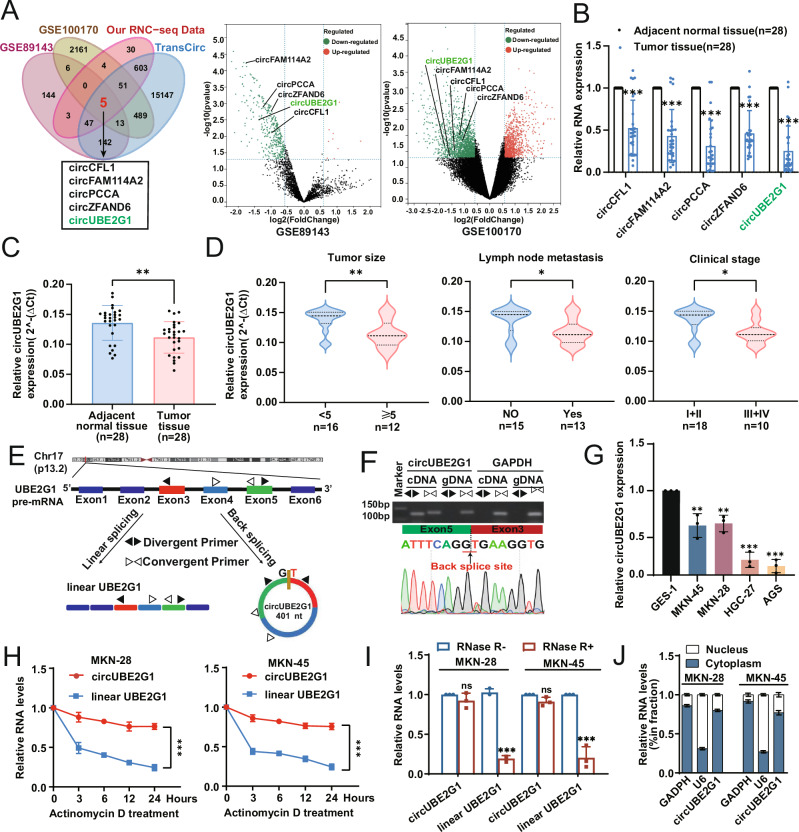
Identification and clinical relevance of circUBE2G1. **A** Venn diagram analysis was performed to identify circRNAs with coding potential by integrating data from the GEO dataset, RNC-seq data, and the TransCirc database (|log2(FC)| ≥ 1, FDR < 0.05). **B** The expression levels of circUBE2G1 in tumor tissues and matched non-tumor tissues from 28 gastric cancer (GC) patients were analyzed using qRT-PCR. **C**, **D** Correlation between circUBE2G1 expression levels and clinicopathological features in 28 GC patients. **E** Schematic representation of circUBE2G1 (hsa_circ_0003239), which arises from exons 3, 4, and 5 of the UBE2G1 gene. **F** Divergent primers specifically detected circUBE2G1 in cDNA but not in gDNA, with GAPDH serving as a negative control. Sanger sequencing confirmed the head-to-tail splicing of circUBE2G1. **G** Expression levels of circUBE2G1 in GC cell lines (MKN-45, MKN-28, HGC-27, and AGS) and normal gastric mucosal epithelial cells (GES-1). Data are presented as means ± SD (*n* = 3). **H** MKN-45 and MKN-28 cells were treated with Actinomycin D, and the relative RNA levels of circUBE2G1 and linear UBE2G1 mRNA were measured at different time points. **I** qRT-PCR analysis of circUBE2G1 and linear UBE2G1 mRNA after RNase R treatment in MKN-45 and MKN-28 cells demonstrated that circUBE2G1 was resistant to RNase R digestion. **J** Nuclear-cytoplasmic fractionation assay revealed that circUBE2G1 was predominantly localized in the cytoplasm. Statistical analysis was performed with Pearson’s correlation analysis. Graph represents mean ± SD; ns, not significant, *P < 0.05, **P < 0.01, and ***P < 0.001.

CircRNA expression profiles from the Gene Expression Omnibus (GEO) repository (accession numbers GSE89143, GSE100170) were systematically analyzed to identify translationally competent circRNAs exhibiting differential expression patterns in gastric cancer. RNC-seq analysis was conducted in GC cells, with the acquired datasets cross-referenced against the Transcirc database to assess circRNA translatability. Overlapping candidates were visualized through Venn diagram analysis. This analysis led to the identification of five circRNAs that were significantly downregulated in GC tissues compared to adjacent normal tissues (Fig. 1A). Quantitative reverse transcription PCR (qRT-PCR) analysis confirmed significant downregulation of circUBE2G1 in 28 paired gastric cancer specimens versus matched adjacent tissues (Fig. 1B). We then examined the relationship between circUBE2G1 expression levels and clinicopathological features of GC patients. The results revealed that lower circUBE2G1 expression correlated with larger tumor size, higher TNM stage, and lymphatic metastasis (Fig. 1C, D and Table S1). These data collectively demonstrate that circUBE2G1 exhibits reduced expression in gastric cancer, with its downregulation correlating with adverse clinicopathological features and serving as an independent prognostic indicator for diminished survival outcomes.

Having established its clinical relevance, we next characterized the molecular architecture of circUBE2G1.CircUBE2G1 is genomically derived from exons 3-5 of the UBE2G1 locus, generating a 401-nucleotide circular RNA transcript through precise head-to-tail splicing (Fig. 1E). To confirm the circular structure of circUBE2G1, divergent and convergent primer pairs were employed for template-specific PCR amplification. Agarose gel analysis revealed exclusive detection of circUBE2G1 in cDNA templates compared to gDNA counterparts from MKN-28 cells, validating back-splicing-mediated circularization. The head-to-tail junction was further verified through Sanger sequencing (Fig. 1F). We next evaluated circUBE2G1 expression in four human GC cell lines (MKN-45, MKN-28, HGC-27, and AGS) and observed significantly decreased levels of circUBE2G1 compared to the normal gastric mucosal epithelial cell line GES-1 (Fig. 1G). To assess the stability and localization of circUBE2G1, we treated cells with actinomycin D (ActD). The half-life of circUBE2G1 was found to be longer than that of linear UBE2G1 mRNA (Fig. 1H). Furthermore, circUBE2G1 demonstrated resistance to RNase R degradation, indicating its high stability (Fig. 1I). Subsequent qRT-PCR analysis of cell fractions revealed that circUBE2G1 was predominantly localized in the cytoplasm, rather than the nucleus (Fig. 1J). Given the significant downregulation of circUBE2G1 in GC and its potential clinical relevance, we sought to investigate the molecular mechanisms underlying its biogenesis, particularly focusing on the regulatory role of splicing factors in circUBE2G1 formation.

Emerging evidence implicates splicing factors in circRNA biogenesis through their binding to conserved intronic motifs flanking circularized exons [14]. To elucidate the molecular mechanisms governing circUBE2G1 regulation, systematic bioinformatic interrogation of UBE2G1 pre-mRNA was performed using RBPsuite, revealing multiple putative splicing factor binding domains. Notably, QKI was prioritized among candidate regulators based on three lines of evidence: firstly, QKI has been documented to possess tumor-suppressive functions with characteristic downregulation across multiple malignancies, including gastric carcinoma [15, 16]; secondly, a robust positive correlation between QKI and circUBE2G1 expression levels was identified in our clinical cohort; and most critically, computational prediction of high-affinity binding motifs within intron 5 (+722, +801) of UBE2G1 pre-mRNA was substantiated (Fig. S1A–C). Subsequent validation through RNA immunoprecipitation (RIP) and RNA pull-down assays definitively demonstrated sequence-specific binding of QKI to the predicted intronic region (Fig. S1D, E). Functional validation revealed that ectopic QKI expression in both GES-1 and MKN-28 cell lines not only elevated QKI protein levels (Fig. S1F) but also significantly enhanced circUBE2G1 biogenesis (Fig. S1G), thereby establishing an essential regulatory axis through which QKI governs circUBE2G1 production.

### CircUBE2G1 inhibits the proliferation and migration of GC cells in vitro

**Fig. 2:**
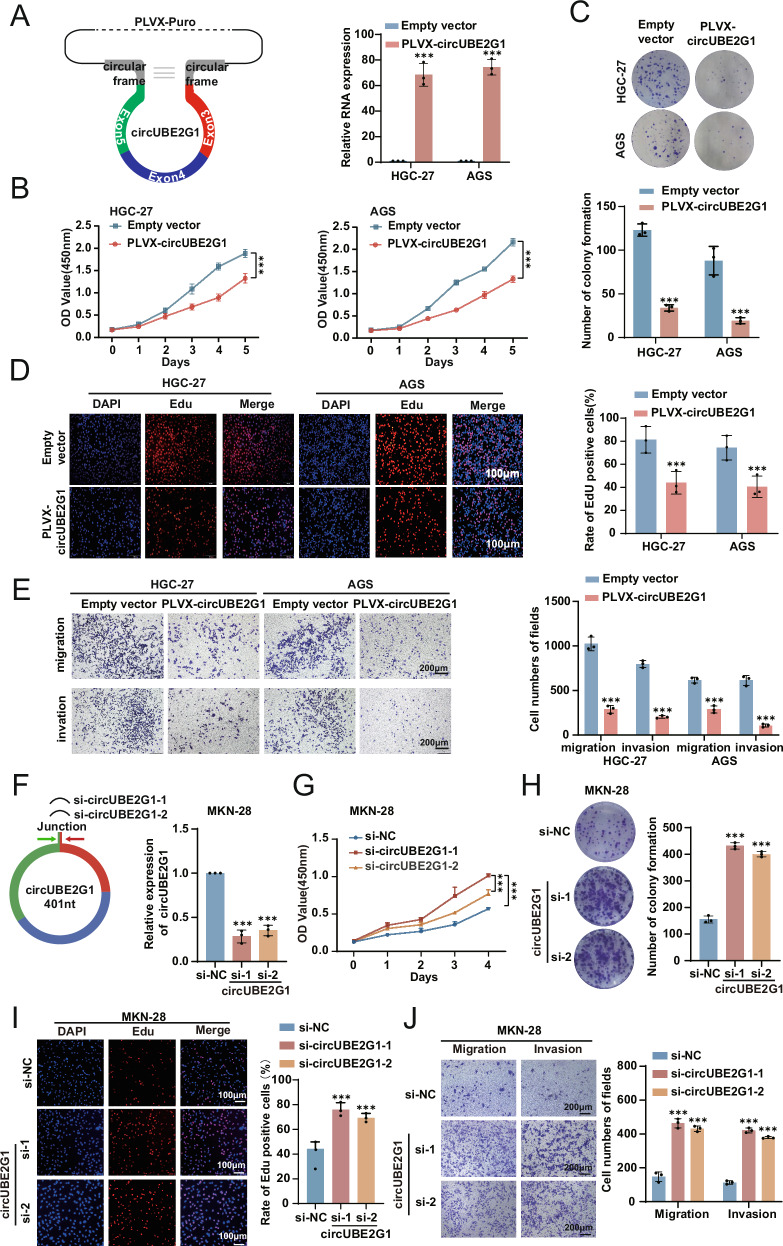
CircUBE2G1 inhibits the proliferation and migration of GC cells in vitro. **A** The efficiency of circUBE2G1 overexpression vectors was verified by qRT-PCR. CCK-8 assay (**B**), Colony formation assay (**C**), and EdU assay **D** were performed to evaluate the proliferation ability of cells after upregulating circUBE2G1 in GC cells. **E** Transwell assay was performed to evaluate the migration ability of cells after upregulating circUBE2G1 in GC cells. **F** Left panel: Diagram of small interfering RNAs (siRNAs) specifically targeting the back-splice junction sequences of circUBE2G1. Right panel: The efficiencies of siRNAs in the MKN-28 cell line were verified by qRT-PCR. CCK-8 assay (**G**), Colony formation assay (**H**), and EdU assay **I** were performed to evaluate the proliferation ability of cells after downregulating circUBE2G1 in GC cells. **J** Transwell assay was performed to evaluate the migration ability of cells after downregulating circUBE2G1 in GC cells. Scale bar: 200 μm. Graphs represent mean ± SD; ***P < 0.001.

Functional interrogation of circUBE2G1 in gastric carcinogenesis was performed through exogenous plasmid-mediated overexpression and siRNA-targeted knockdown strategies. HGC-27 and AGS cells, which exhibit relatively low expression of circUBE2G1, were transfected with a circUBE2G1 expression vector. Overexpression efficiency was confirmed by qRT-PCR (Fig. 2A). CCK-8 assay (Fig. 2B), colony formation (Fig. 2C), EdU assay (Fig. 2D), and transwell migration and matrigel invasion assays (Fig. 2E) demonstrated that circUBE2G1 overexpression significantly suppressed the proliferation, migration, and invasion of GC cells. In contrast, small interfering RNAs (siRNAs) targeting the back-splice region of circUBE2G1 were designed. These siRNAs were transfected into MKN-28 cells, which have relatively high circUBE2G1 expression. qRT-PCR results showed that both siRNA-1 and siRNA-2 effectively reduced circUBE2G1 levels in MKN-28 cells (Fig. 2F). Subsequent CCK-8 assay (Fig. 2G), colony formation (Fig. 2H), and EdU assay (Fig. 2I) revealed that circUBE2G1 silencing significantly enhanced GC cell proliferation. Furthermore, transwell assays (Fig. 2J) indicated that silencing circUBE2G1 promoted the migration of GC cells. These data collectively demonstrate that circUBE2G1 functions as a tumor-suppressive regulator by potently inhibiting the proliferative and migratory capacities of gastric cancer cells.

### CircUBE2G1 encodes a novel tumor suppressor protein circUBE2G1-99aa

**Fig. 3:**
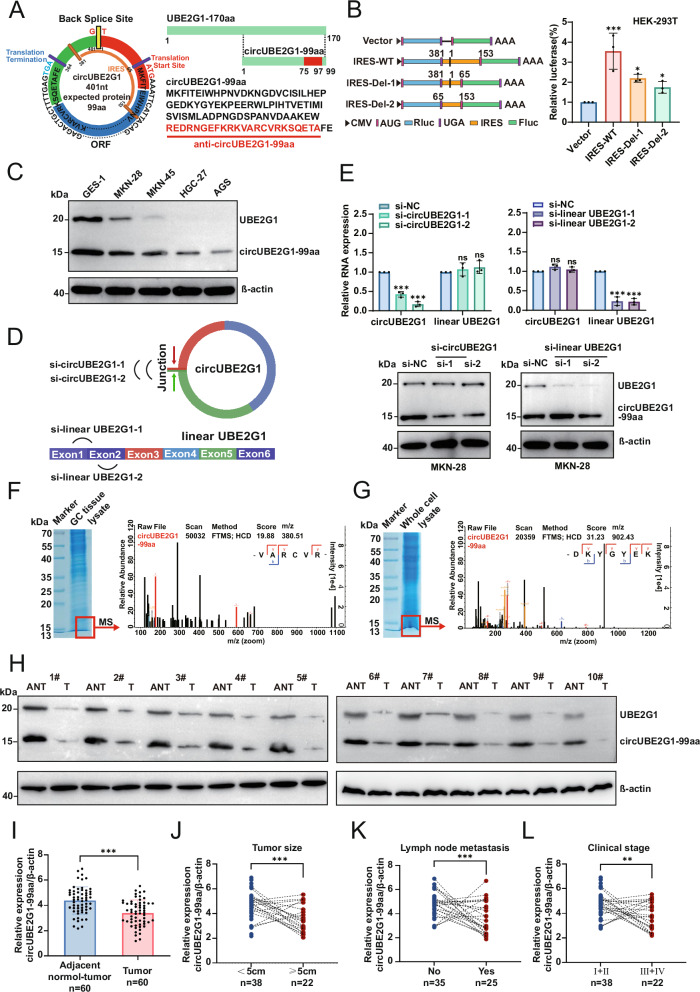
CircUBE2G1 encodes a novel tumor suppressor protein, circUBE2G1-99aa. **A** Left panel: The putative open reading frame ORF in circUBE2G1. Right panel: The sequences of the putative ORF are shown. **B** Left panel: The wild-type or deleted internal ribosome entry site (IRES) was cloned between the Rluc and Luc reporter genes with independent start (AUG) and stop (UGA) codons. Right panel: Relative luciferase activity was tested. **C** UBE2G1-170aa and circUBE2G1-99aa expression were detected in cultured GC cell lines. **D** Illustration of linearUBE2G1 siRNAs and circUBE2G1 siRNAs. **E** Upper panel: The efficiencies of siRNAs in the MKN-28 cell line were verified by qRT-PCR. Lower panel: UBE2G1-170aa and circUBE2G1-99aa expressions were detected using the circUBE2G1-99aa polyclonal antibody in MKN-28 cells treated with indicated siRNAs. Mass spectrometry (MS) identification of circUBE2G1-99aa in GC tissues (**F**) and MKN-28 cells (**G**). **H** UBE2G1-170aa and circUBE2G1-99aa expression were detected in GC tissues and their paired normal tissues. **I** Semi-quantitative analysis of circUBE2G1-99aa in GC tissues. Combined with clinicopathological data analysis of 28 patients with GC, the expression of circUBE2G1-99aa was examined in relation to tumor size (**J**), lymph node metastasis (**K**), and clinical stage (**L**). Graph represents mean ± SD; ns not significant, *P < 0.05, **P < 0.01, and ***P < 0.001.

Based on predictions from the online database circRNADb, an open reading frame (ORF) with an initiation codon (ATG) and internal ribosomal entry sites (IRES) spanning nucleotides 381–153 was identified within the circUBE2G1 sequence. This suggests that circUBE2G1 has the potential to encode a 99-amino-acid (aa) protein, designated circUBE2G1-99aa in this study (Fig. 3A and Table S7). To confirm the activity of the predicted IRES within circUBE2G1, a dual-luciferase assay was performed. The results demonstrated a significant increase in luciferase activity in the wild-type (WT) IRES reporter compared to the deleted IRES reporter (Fig. 3B). This IRES-driven translation led to the synthesis of a novel 99-aa microprotein, sharing the sequence from 72 to 170 aa with WT UBE2G1. To detect this microprotein, we used an antibody targeting the middle region (146–168 aa) of UBE2G1, which also recognizes UBE2G1-170aa. Western blotting revealed down-regulation of endogenous circUBE2G1-99aa in GC cell lines (Fig. 3C).

To rule out the possibility that circUBE2G1-99aa was translated from an alternative start site within the linear UBE2G1 mRNA, siRNAs targeting linear UBE2G1 and circUBE2G1 were used in MKN-28 cells (Fig. 3D). Specific knockdown of circUBE2G1 had no effect on UBE2G1-170aa protein levels, whereas silencing linear UBE2G1 had minimal effects on circUBE2G1-99aa expression (Fig. 3E). Mass spectrometry followed by SDS-PAGE, using protein lysates from GC tissues and MKN-28 cells, confirmed the presence of circUBE2G1-99aa peptide sequences within the predicted molecular weight range (Fig. 3F, G).

To assess the expression profile of circUBE2G1-99aa in clinical GC samples, Western blot analysis was performed on 60 paired GC tissues. Semi-quantitative analysis revealed significantly reduced circUBE2G1-99aa levels in GC tissues compared to normal gastric tissues (Figs. 3H, I and S1H). Furthermore, low expression of circUBE2G1-99aa was correlated with more advanced clinical features, including larger tumor size (Fig. 3J), lymph node metastasis (Fig. 3K), and advanced pathological stage (Fig. 3L and Table S2). Collectively, these findings indicate that circUBE2G1-99aa, encoded by circUBE2G1, is downregulated in GC and its low expression correlates with poor prognosis in GC patients.

### CircUBE2G1-99aa inhibits the proliferation, invasion, and migration abilities of GC in vitro and in vivo

**Fig. 4:**
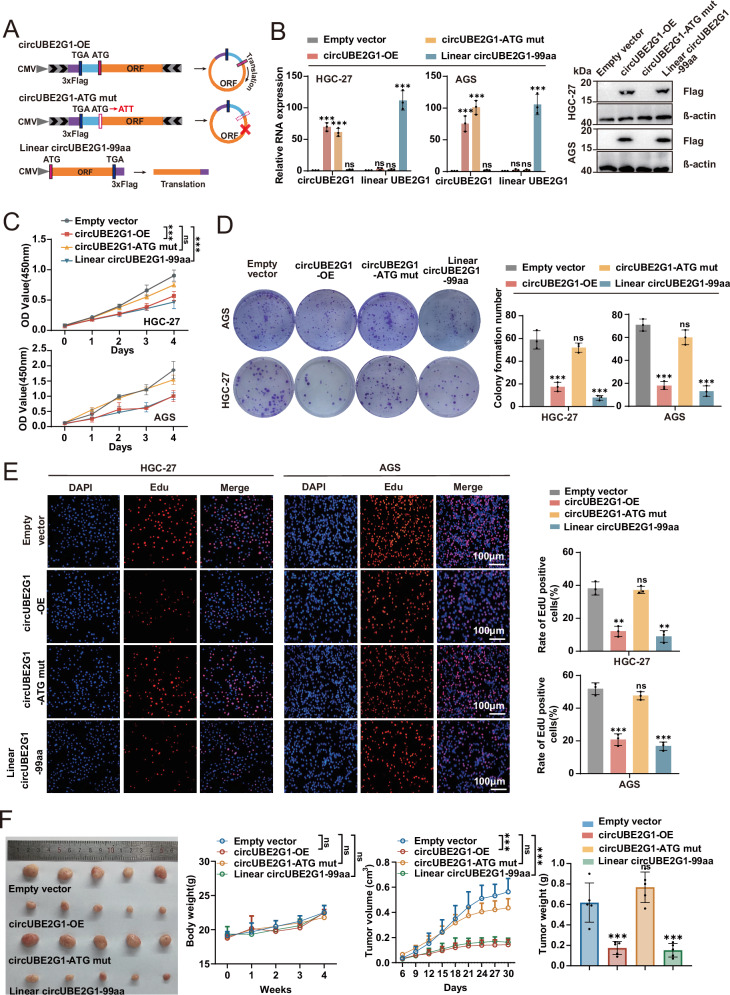
CircUBE2G1-99aa, not circUBE2G1, inhibits the proliferation, invasion, and migration abilities of GC in vitro and in vivo. **A** Schematic diagram of circUBE2G1 overexpression vector (circUBE2G1-OE), start codon mutant vector (circUBE2G1-ATG mut), and linearized circUBE2G1-Flag vector (Linear circUBE2G1-99aa). **B** Left panel, the efficiencies of circUBE2G1 and linearUBE2G1 overexpression vectors were verified by qRT-PCR. Right panel, western blot analysis of Flag-tagged protein expression in cells transfected with the indicated constructs. CCK-8 assay (**C**), colony formation assay (**D**), and EdU assay (**E**) were performed to detect the proliferation ability of cells mentioned above. The number of cells is counted with image (**J**). **F** Subcutaneous xenograft nude mouse models using HGC-27 cells transfected with Empty vector; circUBE2G1-OE, circUBE2G1-ATG mut, or Linear circUBE2G1-99aa were established with tumor growth curves (*n* = 5, each group). Scale bar: 200 μm. Graph represents mean ± SD; ns not significant, **P < 0.01, and ***P < 0.001.

Functional characterization of circUBE2G1-99aa was achieved through transfection of four distinct Flag-tagged constructs into HGC-27 and AGS cells: (1) Empty vector control; (2) circUBE2G1-OE: CMV promoter-driven expression vector containing Flag-tagged circUBE2G1 flanked by inverted repeats for circularization; (3) circUBE2G1-ATGmut: ATG-to-ATT start codon mutation in the Flag-tagged circUBE2G1 backbone; (4) Linear-99aa: CMV-driven linear construct expressing Flag-tagged circUBE2G1-99aa without flanking inverted repeats (Fig. 4A). As expected, circUBE2G1-99aa (15 kDa) encoded by circUBE2G1 was absent in the cells transfected with the Empty vector or ATG mutant plasmid (Fig. 4B). CCK-8 assay, colony formation and Edu assays revealed that the proliferation of GC cells was significantly inhibited following transfection with circUBE2G1-OE or Linear circUBE2G1-99aa. In contrast, transfection with circUBE2G1-ATG mut plasmid had no effect on cell proliferation (Fig. 4C, D, E). Additionally, over-expression of circUBE2G1-99aa reduced the invasion and migration abilities of GC cells, whereas circUBE2G1-ATG mut had no impact on these cellular functions (Fig. S1I).

To further assess whether circUBE2G1-99aa exerts a tumor-suppressing effect in vivo, a xenograft mouse model was established by subcutaneously injecting HGC-27 cells (*n* = 5 for each group). Tumor growth rates and weights were significantly lower in the circUBE2G1-OE and Linear circUBE2G1-99aa groups compared to the Empty vector and circUBE2G1-ATG mut groups (Fig. 4F). In summary, these results demonstrate that the inhibitory effects of circUBE2G1 on the malignant biological behaviors of GC cells are primarily mediated by the protein encoded by circUBE2G1, circUBE2G1-99aa.

### CircUBE2G1-99aa regulates GC cell glycolysis by binding to ENO1

**Fig. 5:**
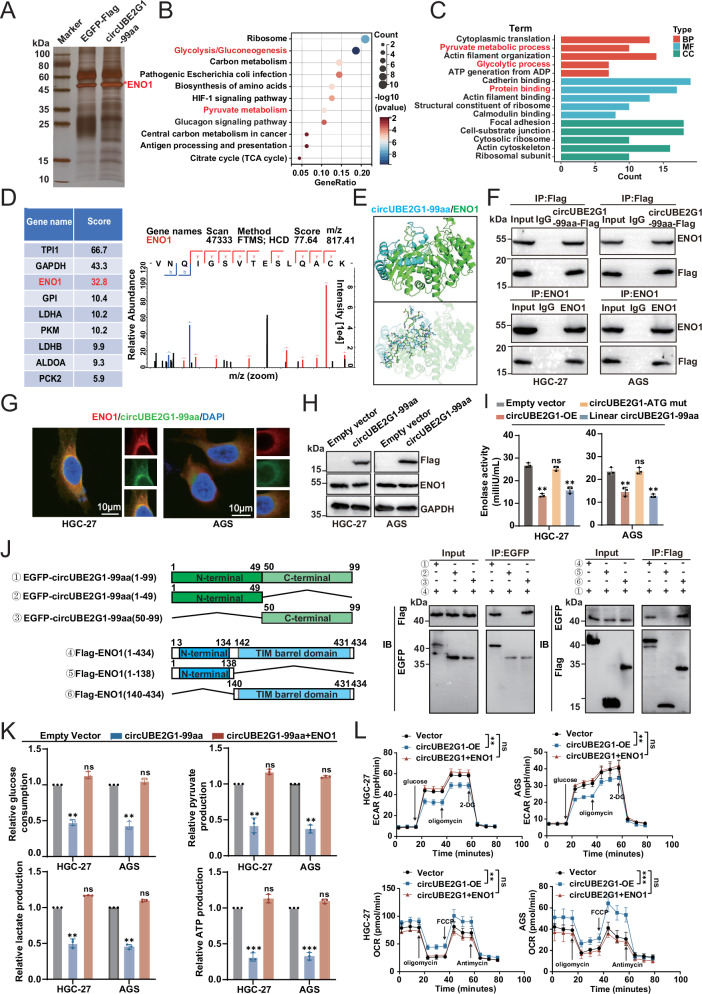
CircUBE2G1-99aa regulates GC cell glycolysis by binding to ENO1. **A** In the left panel, Total protein from circUBE2G1-99aa plasmid-transfected HGC-27 cells was separated via SDS-PAGE. Arrows show different bands between IgG and Flag. ENO1 was identified by LC/LC-MS in circUBE2G1-99aa protein complex. Kyoto Encyclopedia of Genes **B** and Genomes pathway analysis **C** showed the significantly affected signaling pathway on circUBE2G1-99aa overexpression in GC cells. **D** Table presenting scores of glycolytic-related proteins identified by mass spectrometry to interact with circUBE2G1-99aa. **E** Illustration depicting the predicted binding mode of circUBE2G1-99aa with ENO1, as modeled using AlphaFold and visualized in PyMol software. **F** Mutual interaction of ENO1 and Flag-circUBE2G1-99aa was determined by IP. **G** Flag-tagged circUBE2G1-99aa was transfected into HGC-27 and AGS cells, and immunofluorescence was performed using anti-Flag and anti-ENO1 antibodies. Scale bar, 10 μm. **H** Overexpress circUBE2G1-99aa in GC cell lines HGC-27 and AGS, then quantify ENO1 mRNA and protein expression levels using qRT-PCR and Western blot, respectively. **I** Overexpression of circUBE2G1-99aa in GC cells HGC-27 and AGS, followed by measurement of ENO1 enzyme activity. **J** Left panel, Schematic illustration of ENO1 domain truncation and the circUBE2G1-99aa truncation diagram. Right panel, Co-IP validation of the binding region between circUBE2G1-99aa and ENO1. **K** Glucose consumption, pyruvate, lactate production, and ATP generation were measured in HGC-27 and AGS cells divided into three groups: Empty vector, circUBE2G1-99aa, and circUBE2G1-99aa + ENO1. **L** Extracellular acidification rates and cellular oxygen consumption rates were determined using the Seahorse assay for each group. Graph represents mean ± SD; ns not significant, **P < 0.01, and ***P < 0.001.

To elucidate the molecular mechanism underlying the regulation of GC cell biology by circUBE2G1-99aa, we investigated proteins interacting with circUBE2G1-99aa in HGC-27 cells. Using immunoprecipitation coupled with mass spectrometry, we identified 93 proteins associated with circUBE2G1-99aa (Fig. 5A). KEGG pathway and GO enrichment analyses revealed that these interacting proteins were predominantly involved in glycolysis/gluconeogenesis, carbon metabolism, pyruvate metabolism, and the tricarboxylic acid cycle (TCA cycle) (Fig. 5B, C). Notably, enolase 1 (ENO1), which ranked highly in the mass spectrometry results, drew our attention (Fig. 5D). ENO1, a key enzyme in the glycolytic pathway, plays a critical role in regulating glycolysis and has been recognized as a promising therapeutic target in cancer [17]. Therefore, ENO1 was selected as a candidate interactor of circUBE2G1-99aa. Structural predictions using AlphaFold and molecular docking simulations with PyMOL suggested a potential interaction between circUBE2G1-99aa and ENO1 (Fig. 5E). To validate this interaction, Co-IP assays in both directions and immunofluorescence assays were performed, confirming that circUBE2G1-99aa binds to ENO1, with both proteins primarily co-localizing in the cytoplasm (Fig. 5F, G). While dysregulation of ENO1 expression or catalytic activity has been demonstrated to disrupt glucose homeostasis and enhance lactate biosynthesis, consequently facilitating malignant progression in neoplastic cells, the precise molecular mechanisms through which circUBE2G1-99aa orchestrates the ENO1-dependent glycolytic axis in gastric carcinogenesis remain to be systematically delineated [18–20].

### CircUBE2G1-99aa regulates the glycolytic pathway by influencing ENO1 activity

To investigate the relationship between circUBE2G1-99aa and ENO1, we examined the effect of circUBE2G1-99aa on ENO1 expression in HGC-27 and AGS cells. We observed no significant changes in the transcriptional or protein levels of ENO1 following circUBE2G1-99aa overexpression (Figs. 5H and S1J). Enzymatic profiling demonstrated that genetic ablation of circUBE2G1-99aa resulted in enhanced ENO1 activity, which was effectively rescued to baseline levels upon reintroduction of wild-type circUBE2G1-99aa. In contrast, reconstitution with the ATG-mutant circUBE2G1-99aa (ATG → ATT) failed to restore ENO1 activity, suggesting that circUBE2G1-99aa regulates ENO1 via modulation of its enzymatic function (Fig. 5I). To delineate the structural determinants governing the circUBE2G1-99aa/ENO1 interaction, systematic co-immunoprecipitation assays were conducted using truncation variants of both proteins. The C-terminal region of circUBE2G1-99aa (residues 50-99) demonstrated robust binding to full-length ENO1, while conversely, the catalytically essential TIM barrel domain of ENO1 (residues 140-434) specifically co-precipitated with circUBE2G1-99aa (Fig. 5J). These findings confirm that circUBE2G1-99aa binds specifically to the TIM barrel catalytic domain of ENO1. The TIM barrel domain, commonly found in enzymes involved in sugar metabolism, directly participates in substrate binding and catalytic reactions, thereby enhancing the enzymatic activity. To assess whether circUBE2G1-99aa affects aerobic glycolysis in GC cells by modulating ENO1 enzyme activity, circUBE2G1-99aa or mutants were first overexpressed in GC cells, and glucose utilization and the production of pyruvate, lactate, and ATP (Fig. S1K) were measured. The Seahorse assay was also conducted to quantify the ECAR and OCR in these cells (Fig. S1L). These results indicated that the expression level of circUBE2G1-99aa in gastric cancer cells was significantly and negatively correlated with cellular glucose uptake rate, extracellular acidification rate (ECAR), pyruvate and lactate output, and ATP production. Next, overexpression of ENO1 in circUBE2G1-99aa high-expressing GC cells was performed for glucose metabolism phenotyping studies. Our results revealed that ENO1 overexpression effectively alleviated the inhibitory effect of circUBE2G1-99aa on glycolytic flux (Fig. 5K, L).

### CircUBE2G1-99aa targets ENO1 to regulate PI3K/AKT signaling and inhibit glycolysis in GC cells

**Fig. 6:**
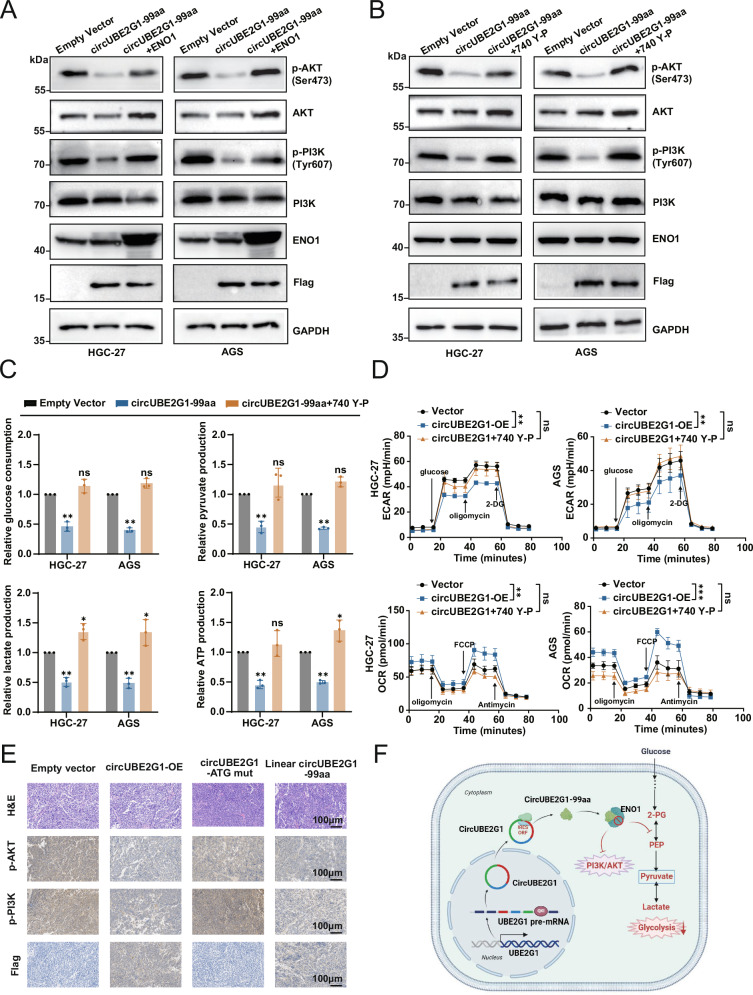
CircUBE2G1-99aa targets ENO1 to regulate PI3K/AKT signaling and inhibit glycolysis in GC cells. **A** An evaluation of PI3K/AKT signaling pathway-related protein level alterations by Western blot in HGC-27 and AGS cells stably overexpressing circUBE2G1-99aa following ENO1 overexpression. **B** Investigation of PI3K/AKT signaling pathway protein levels via Western blot in circUBE2G1-99aa-stably overexpressing HGC-27 and AGS cells treated with the PI3K/AKT pathway activator 740 Y-P. **C**, **D** Measurement of glucose consumption, pyruvate, lactate production, ATP levels, and ECAR and OCR using Seahorse analysis in HGC-27 and AGS cells divided into Empty vector, circUBE2G1-99aa, and circUBE2G1-99aa + 740 Y-P groups. **E** Subcutaneous xenograft tumor models established in BALB/c Nude mice using HGC-27 cell lines (Empty vector, circUBE2G1-OE, circUBE2G1-ATG mut, and Linear circUBE2G1-99aa), followed by H&E staining and immunohistochemical detection of p-PI3K (Tyr607) and p-AKT (Ser473) expression levels in tumor tissues. Scale bar: 100 μm. **F** Schematic illustration of the mechanism by which circUBE2G1-99aa regulates the ENO1/PI3K/AKT axis to inhibit glycolysis in GC cells. Graph represents mean ± SD; ns not significant, *P < 0.05, **P < 0.01, and ***P < 0.001.

Previous studies have implicated ENO1 in tumor progression via the PI3K/AKT pathway [21, 22]. Here, we used western Blot analysis to assess ENO1’s impact on PI3K/AKT signaling in stable GC cells overexpressing circUBE2G1-99aa, demonstrating restored phosphorylation levels of PI3K and AKT without altering total protein levels (Fig. 6A). To investigate whether circUBE2G1-99aa’s effects on GC cells are PI3K/AKT-dependent, we activated the pathway using the PI3K agonist 740 Y-P (20 µg/mL), which, akin to ENO1 overexpression, also revived PI3K and AKT phosphorylation (Fig. 6B). Analyses of glucose, pyruvate, lactate, ATP levels, ECAR, and OCR revealed that 740 Y-P significantly reinstated glycolytic capacity in circUBE2G1-99aa-overexpressing cells (Fig. 6C, D). Immunohistochemistry of tumor tissues confirmed reduced levels for p-PI3K and p-AKT, indicative of attenuated PI3K/AKT activation following circUBE2G1-99aa up-regulation (Fig. 6E). Collectively, these findings position ENO1 as a crucial upstream regulator of the PI3K/AKT pathway in GC progression, highlighting its essential role in circUBE2G1-99aa-mediated mechanisms.

## Discussion

Circular RNAs (circRNAs) have emerged as pivotal regulators in cancer biology, with diverse functional roles that extend beyond their traditional classification as non-coding RNAs6. While a substantial proportion of circRNAs are known to function as microRNA sponges or protein scaffolds, recent discoveries have unveiled a subset of circRNAs that encode functional peptides or proteins with significant implications in cancer progression [23, 24]. Notable examples include circHGF, which encodes C-HGF to activate c-MET signaling and promote glioblastoma growth [25], and circZKSCAN1, which suppresses hepatocellular carcinoma through its interaction with FBXW7 and mTOR, thereby inhibiting the PI3K/AKT pathway [26]. In the present study, we identified circUBE2G1 as a novel circRNA in gastric cancer (GC) that encodes a functional protein, circUBE2G1-99aa. Our findings demonstrate that circUBE2G1-99aa is downregulated in GC and exerts tumor-suppressive effects by inhibiting glycolysis and cell proliferation.

The biogenesis of circRNAs, which are characterized by their covalently closed structure, is tightly regulated by specific RNA-binding proteins (RBPs) and cis-acting elements [27]. Previous research has established that RBPs such as QKI and Mbl play crucial roles in circRNA formation through their binding to specific sequences [28–30]. In this investigation, we employed RNA immunoprecipitation (RIP) and RNA pull-down assays to demonstrate that QKI binds to the flanking sequences of circUBE2G1, thereby promoting its biogenesis. This interaction is essential for the production of circUBE2G1-99aa in GC cells.

To elucidate the functional mechanisms of circUBE2G1-99aa in GC, we investigated its interaction with ENO1, a key glycolytic enzyme. ENO1 is widely recognized for its role in promoting the Warburg effect in cancer cells, and its overexpression is associated with poor prognosis in various cancers, including GC [31–34]. While overexpression of circUBE2G1-99aa did not alter ENO1 mRNA or protein levels, structural analysis revealed that circUBE2G1-99aa binds to the TIM barrel catalytic domain of ENO1, suggesting a potential regulatory effect on ENO1’s enzymatic activity. This interaction likely contributes to the suppression of glycolysis in GC cells, a mechanism that requires further exploration.

Additionally, we examined the impact of circUBE2G1-99aa on the PI3K/AKT pathway, a critical regulator of cellular metabolism and survival [35–37]. Our data indicate that overexpression of circUBE2G1-99aa reduces the phosphorylation of PI3K and AKT, signifying inhibition of this pathway. Notably, ENO1 overexpression was found to counteract this effect, providing further evidence that circUBE2G1-99aa modulates glycolysis in GC cells through the ENO1/PI3K/AKT axis.

## Conclusion

Through integrated multi-omics analysis and experimental validation, circUBE2G1-99aa was identified as a previously uncharacterized circRNA-encoded protein in gastric cancer. The study demonstrates that circUBE2G1-99aa is downregulated in GC and suppresses tumor progression by binding to the catalytic TIM barrel domain of ENO1, thereby inhibiting its enzymatic activity and glycolysis (Fig. 6F). However, the direct molecular mechanism by which circUBE2G1-99aa suppresses ENO1 activity requires further validation. Future studies should focus on validating this regulatory axis and exploring the broader functional networks of circUBE2G1-99aa in GC pathogenesis.

## Methods and materials

### Human GC tissues and normal tissues

Matched primary GC tissues and adjacent nontumor tissues were collected from 60 GC patients at the Affiliated Suzhou Hospital of Nanjing Medical University (surgery performed in December 2021), comprising 38 males and 22 females. This study was approved by the Medical Ethics Committee of the Affiliated Suzhou Hospital of Nanjing Medical University, and all participants priors provided written informed consent. Clinicopathological features include age, gender, tumor size, tumor site, lymph node metastasis, and TNM stage (according to the American Joint Committee on Cancer Classification, AJCC). The inclusion criteria were defined as the certain diagnosis of GC. Also, patients who had received any treatment such as chemotherapy, radiotherapy, and biological medication (monoclonal antibodies) before the sampling were excluded from the study.

### Cell culture

The human gastric mucosal cell line GES-1, the GC lines MKN-28, MKN-45, HGC-27, and AGS, and the human embryonic kidney epithelial cell line HEK-293T were purchased from the National Collection of Authenticated Cell Cultures (Shanghai, China) and authenticated by STR before shipping. The cells were tested for negative mycoplasma contamination using Mycoplasma Detection Kit (Sigma-Aldrich, MP0050, Missouri, USA). All cells, except AGS cells and HGC-27 cells, were cultured in DMEM, and AGS cells and HGC-27 cells were cultured in RPMI 1640. All media were supplemented with 10% fetal bovine serum (FBS) in a humidified atmosphere of 5% CO_2_ at 37 °C. No mycoplasma contamination was detected in all cells.

### Quantitative reverse transcription PCR assay (qRT–PCR)

Total RNAs were extracted from tissues and cells using TRIzol reagent. RNA concentration and quality were measured using a NanoDrop2000 Spectrophotometer. Then, 1 μg RNA was subjected to reverse transcription using the One Step TB Green® PrimeScript™ RT-PCR Kit (TaKaRa, Liaoning, China) on the ABI 7500 Fast Real-Time PCR system. The primers are given in Table S6. The results were calculated using the 2^−ΔΔCt^ relative quantification method, and β-actin served as an endogenous control.

### RNase R digestion and actinomycin D treatment

RNase R digestion and actinomycin D treatment were both performed to confirm the stability of circUBE2G1 in GC cells. For RNase R digestion, 20 μg total RNA was incubated with 20 U/μl RNase R (GEENSEED, R0301) for 15 min at 37 °C. For actinomycin D treatment, cells were treated with 2 mg/ml actinomycin D (NobleRyder, Beijing, China) and harvested after incubation for 0, 4, 8, 12, and 24 h. After treatment with RNase R or actinomycin D, qRT–PCR assays were conducted to determine the expression of circUBE2G1 and linear UBE2G1.

### Nuclear and cytoplasmic extraction

Nuclear and cytoplasmic fractions were isolated using the PARIS™ kit (Thermo Fisher, Waltham, MA). Briefly, MKN-28 and MKN-45 cells were lysed by Cell Fractionation Buffer for 10 min on ice. Then, the supernatants were collected as the cytoplasmic fractions after centrifugation at 4 °C and 500 × *g* for 3 min. Finally, the pellets were lysed by Cell Disruption Buffer to collect the nuclear fractions.

### RNA Immunoprecipitation assay (RIP)

EZ-Magna RIP™ RNA-Binding Protein Immunoprecipitation Kit (Millipore, Billerica, MA) was utilized for RIP assay. One hundred microliters lysates of GES-1 cells and MKN-28 cells were incubated with 50 μl magnetic beads coupled with 5 μg anti-QKI overnight at 4 °C. Rabbit IgG antibody (Santa Cruz Cat# sc-2025) was used as the negative control. The immunoprecipitated RNA was isolated by proteinase K and analyzed by qRT–PCR assays. The primers are given in Table S3.

### RNA pull-down assay

For the RNA binding protein (RBP)–intron interaction, the intron RNAs were obtained using in vitro transcription by T7 RNA polymerase (Ambion Life, USA) and purified by RNeasy Plus Mini Kit (QIAGEN, USA). Then, we conducted the pull-down assay by biotin-labeled RNAs and Streptavidin magnetic beads (Life Technologies). Western blotting assay was utilized to detect the proteins pulled down.

### Cell transfection

Cells were seeded into 24-well plates and transfected with plasmids using Lipofectamine 3000 reagent (Life Technologies, Carlsbad, CA) based on the experimental groupings when cell confluence reached 50–70%. For each well, 0.75 μl Lipofectamine 3000 reagent was diluted with 25 μl Opti-MEM Medium (Thermo Fisher, Waltham, MA). Five hundred nanograms plasmid DNA were diluted with 25 μl Opti-MEM Medium and 1 μl P3000 reagent. Then, the diluted DNA was incubated with diluted Lipofectamine 3000 reagent for 10 min at room temperature. Finally, the mixture was added into the well and incubated with cells for 48 h at 37 °C. The stably transfected cells were screened by neomycin or puromycin, and the transfection efficiency was detected by qRT–PCR and western blot assays.

The siRNAs against circUBE2G1 (si-circUBE2G1: site #1, 5′- TCACTTATTTCAGGTGAAG-3′; site #2, 5′- ACTTATTTCAGGTGAAGGT -3′) were synthesized by GenePharma, the siRNAs against UBE2G1 (si-UBE2G1: site #1, 5′-CAGGTTTAATAGATGACAA-3′; site #2, 5′-CCTCCAGATACACTTTATG-3′) were synthesized by GeneChem, and their corresponding empty plasmids (si-circUBE2G1-NC, and si-UBE2G1-NC) were constructed as the negative control. The plasmids with QKI full-length sequence (QKI-OE), circUBE2G1 full-length sequence (circUBE2G1-OE) or mutant ATG (circUBE2G1-ATG mut), circUBE2G1-99aa full-length sequence (Linear circUBE2G1-99aa), ENO1 full-length sequence, and their corresponding empty plasmids were constructed by GenePharma.

### Lentiviral production and establishment of stable cell lines

Lentiviral vectors expressing circUBE2G1-OE, circUBE2G1-ATG mut, and Linear circUBE2G1-99aa were co-transfected with the packaging vectors psPAX2 (Addgene) and pMD2G (Addgene) into HEK293T cells for lentivirus production using Lipofectamine 3000 (Thermo Fisher Scientific) according to the manufacturer’s instructions. To establish stable cell lines, GC cells were transduced by using the above lentiviruses with polybrene (8 mg ml-1, Sigma). After incubating for 72 h, cells were selected with 2 mg ml−1 puromycin for 3 days.

### CCK-8 assay

The cell proliferation ability was monitored by the Cell Counting Kit-8 assay (Beyotime Biotechnology, Shanghai, China). A total of 1 × 103 cells were cultured into 96-well plates and measured absorbance values at 450 nm every day by the automatic microplate reader (BioTek, Winooski, VT, USA). The experiment was performed in triplicate.

### 5-Ethynyl-2′-deoxyuridine (EdU) assay

The treated cells were first plated into 96-well plates (3 × 10^4^/well) and cultured for 24 h before the adjunction of EdU (50 μmol/L). Next, cells were fixed in 4% formaldehyde for 2 h and permeabilized with 0.5% TritonX-100 for 10 min at room temperature. 1×Apollo reaction solution (400 μl) was added to react with the EdU (Beyotime Biotechnology, Shanghai, China) for 30 min, and DAPI (400 μl) was added for 30 min to stain the nucleus after washing with PBS three times. Finally, a Nikon microscope (Nikon Japan) was used to capture the images of cells. The experiment was performed in triplicate.

### Colony formation assay

The different transfections of cells were plated into 6-well plates with complete medium to culture for 2 weeks. After that, the numbers of colony formations were counted after staining with crystal violet. The experiment was performed in triplicate.

### Transwell assay

A total of 4 × 10^4^/well-transfected cells in 250 μl fresh medium with FBS were plated into the upper chamber, including a Matrigel-coated or uncoated membrane. After that, the complete medium (750 μl) was supplied to the lower section. The cells that invaded or migrated to the low membrane were stained with 0.1% crystal violet (Beyotime Biotechnology, Shanghai, China) after 48 h incubation. The experiment was performed in triplicate.

### Western blot assay

Total proteins were extracted from tissues and cells using RIPA lysis buffer (Beyotime, Shanghai, China). The BCA kit (Beyotime Biotechnology, Shanghai, China) was utilized to measure the concentration of protein was quantified by using. After the electrophoresis, the PVDF membranes were used. We incubated the membranes with different specific primary antibodies at 4 °C overnight after blocking them with 5% non-fat milk in a shaker for 2 h. On another day, the corresponding secondary antibodies were used at room temperature for 2 h. Finally, Proteins were identified using the BeyoECL Star Kit (Beyotime, Shanghai, China) and captured by the MicroChemi chemiluminescent imaging system (DNR, Jerusalem, Israel). ImageJ software was used for analyzing the bands, and β-actin was used as an endogenous control. The product numbers of antibodies were listed: QKI (A22723, ABclonal, 1:1000), ENO1 (A1033, ABclonal, 1:1000), AKT (AP0059, Bioworld, 1:1000), p-AKT phosphorylated Ser473 (9271, Cell Signaling Technology, 1:1000), PI3K (ab70598, Abcam, 1:1000), p-PI3K phosphorylated Tyr607, β-tubulin (ab6046, Abcam, 1:500), GAPDH, and β-actin were used as internal controls. The experiment was performed in triplicate.

### Immunofluorescence assay (IF)

Cells seeded on glass slides were fixed in 4% paraformaldehyde for 30 min and permeabilized with 0.2% TritonX-100 for 20 min and then blocked with 5% BSA for 2 h at room temperature. Next, the cell slides were incubated with primary antibodies at 4 °C overnight. Then, the cell slides were washed with PBST three times and incubated with fluorescent-conjugated secondary antibodies, Goat anti-rabbit Alexa Fluor 488 or Goat anti-rabbit Cy3 (Beyotime, Shanghai, China), for 2 h at room temperature away from the light. Finally, the nuclei were counterstained with DAPI for 5 min. Fluorescence was visualized under laser confocal microscopy.

### Metabolism assay

Pyruvate, lactate, and ATP production and intracellular glucose uptake were measured using pyruvate, lactate, and ATP assay kits (Nanjing Jiancheng, Jiangsu, China) according to the manufacturer’s instructions, respectively.

### Determination of extracellular acidification rate (ECAR) and oxygen consumption rate (OCR)

ECAR and OCR were determined using a Seahorse XP96 extracellular flux analyzer (Seahorse Bioscience, USA) according to the methods of Ling Li et al. ECAR and OCR were obtained by the Seahorse XP glycolytic Stress Test Kit and the Seahorse XP Cellular Mitochondrial Stress Test Kit (Agilent Technologies, USA), respectively. The glycolytic level, glycolytic capacity, basal OCR, and maximum OCR were calculated according to the test results.

### Dual-luciferase reporter assay

The reporter vector constructions were carried out by inserting full-length, truncated, or deleted IRES into dual-luciferase reporter vectors. 293T cells were transfected with constructed reporter vectors, and the relative luciferase activities were detected 48 h after transfection using the Dual-Luciferase® Reporter Assay System Kit (Promega, Madison, WI) following the manufacturer’s protocol.

### Enolase activity

Enolase activity was determined using the Enolase Activity Assay Kit according to the manufacturer’s instructions (Sigma, Germany, MAK178-1KT). In brief, the samples of cell lysates were mixed with reaction buffer and incubated at 25 °C. After 5–10 min, an initial measurement of OD value was taken at a wavelength of 570 nm and was followed by measurement every 2–3 min until the OD value of the most active sample was greater than the value of the highest standard for getting the final measurement. Enolase activity was calculated using an equation described previously.

### Co-immunoprecipitation assay (Co-IP)

Co-IP assays were performed using the Pierce Co-immunoprecipitation (Co-IP) Kit (Thermo Fisher, Waltham, MA) following the manufacturer’s instructions. Cell lysates were prepared and incubated with AminoLink Plus Coupling Resin immobilized primary antibody overnight at 4 °C. Then, the samples were washed three times with 200 μl Wash Buffer and eluted with Elution Buffer for 5 min. The eluates were finally analyzed by Western blot assays.

### Mass spectrometry analysis

SDS-PAGE gel electrophoresis separated proteins, and the targeted protein bands were excised from the gel. After elution, reduction, and alkylation, the proteins were digested at 37 °C overnight. Peptides were collected, desalted, and analyzed by times of pro-mass spectrometer (Bruker, Bremen, Germany). Sequence and site identification were analyzed using the NCBI nonredundant protein database with Mascot Daemon.

### Tumor xenografts in nude mice

All animal procedures were approved by the Institutional Animal Care and Use Committee of Nanjing Medical University (IACUC) (permit number IACUC-2407017). The 5-week-old female BALB/c nude mice were used. The stably transfected HGC-27 cells were selected and divided into four groups (*n* = 5 each group). For subcutaneous xenografts, 3 × 10^6^ cells were subcutaneously injected into the right flank of each nude mouse. Tumor volumes were measured every 4 days and calculated by formula: volume (mm^3^) = length × width2/2. The mice were sacrificed at the end of the experiments, and the subcutaneous tumors were weighed and photographed.

### Statistical analysis

All experimental data were indicated as mean ± SD. GraphPad Prism 5.01 was used for statistical analysis. Comparison between groups was analyzed by Student’s *t* test, one-way ANOVA, or two-way ANOVA. Statistical significance was determined by *P* value < 0.05.

## Supplementary information

Supplementary Table

Supplementary figure and figure legend

WB original drawing+marker

## Data Availability

The data that support the findings of this study are available from the corresponding author upon reasonable request.
