# Supplementary material for: A novel protein encoded by circUBE2G1 suppresses glycolysis in gastric cancer through binding to ENO1
Source: Cell Death Discov. 2025 Jul 29;11:350. doi: 10.1038/s41420-025-02644-0 (PMC12307642; doi:10.1038/s41420-025-02644-0)
Supplement: Supplementary file 1 — Supplementary Table [file 41420_2025_2644_MOESM1_ESM.pdf]

Table of contents

Contents	Page
Table S1. The clinical relevance of circUBE2G1 expression in gastric cancer	2
Table S2. The clinical relevance of circUBE2G1-99aa expression in gastric cancer	3
Table S3. Five primer pairs of QKI used in RIP analyses	4
Table S4. Two circUBE2G1 siRNAs used in circUBE2G1 silencing	4
Table S5. Two linear UBE2G1 siRNAs used in linear UBE2G1 silencing	4
Table S6. Primers used in this study	5
Table S7. Sequences of circUBE2G1 RNA and protein	6
Table S8.Candidate interacting proteins of circUBE2G1-99aa identified by proteomic profiling	7

Table S1. The clinical relevance of circUBE2G1 expression in gastric cancer

Variables	N (%)	circUBE2G1[a]		*p-value*[b]
		High	Low	
**Gender**				0.6891
**Male**	19 (68%)	11	8	
**Female**	9(32%)	4	5	
**Age(years)**				0.5238
**＜55**	2(7%)	2	0	
**≥55**	26(93%)	16	10	
**Tumor size(cm)**				0.0060**
**＜5**	16(57%)	13	3	
**≥5**	12(43%)	3	9	
**Lymph node metastasis**				0.0115*
**No**	15(53%)	11	4	
**Yes**	13(47%)	3	10	
**TNM stage**				0.0054**
**I+II**	18(64%)	14	4	
**III+IV**	10(36%)	2	8	

[a] Fold change (FC) (tumor tissues relative to normal tissues) Fold change is greater than or equal to 2.0 for high expression, and less than 2.0 for low expression.

[b] *$P<0.05$，**$P<0.01$ was considered significant (Chi-square test between 2 groups)

Table S2. The clinical relevance of circUBE2G1-99aa expression in gastric cancer

Variables	N (%)	circUBE2G1-99aa[a]		*p-value*[b]
		High	Low	
**Gender**				0.7397
**Male**	38 (63%)	17	21	
**Female**	22(37%)	10	12	
**Age(years)**				0.5230
**＜55**	19(32%)	8	11	
**≥55**	41(68%)	15	26	
**Tumor size(cm)**				0.0004***
**＜5**	38(58%)	30	8	
**≥5**	22(42%)	7	15	
**Lymph node metastasis**				0.0008***
**No**	35(58%)	29	6	
**Yes**	25(42%)	10	15	
**TNM stage**				0.0012**
**I+II**	38(58%)	29	9	
**III+IV**	22(42%)	10	12	

[a] Fold change (FC) (tumor tissues relative to normal tissues) Fold change is greater than or equal to 2.0 for high expression, and less than 2.0 for low expression.

[b]***P<0.01 ， ***P<0.001 was considered significant (Chi-square test between 2 groups)

Table S3. Five primer pairs of QKI used in RIP analyses

pair #a	F	GCAGCCCTGTTTGAAAAACTAG
	R	GGCTTGAGGGAATAGAGACTGG
pair #b	F	TGATCTAGCATCCTGATCACTTGG
	R	GCTAGTCTCAAACCCCTGATCTC
pair #c	F	GCTAAGGAGAACTTTCAAGATCGTC
	R	GTTGTCTATCCTTAAGCCATTGG
pair #d	F	GTGGTGTGAAAGAGCAGTAGAAG
	R	CAAGCCAGCAGATGCATCTTAC
pair #e	F	GGAAGTAAATGCCTCAGTAAGTACC
	R	CTGGACAAGCTAGCATACTATACTC

Table S4. Two circUBE2G1 siRNAs used in circUBE2G1 silencing

siRNA #1	TCACTTATTTCAGGTGAAG
siRNA #2	ACTTATTTCAGGTGAAGGT

Table S5. Two linear UBE2G1 siRNAs used in linear UBE2G1 silencing

siRNA #1	CAGGTTTAATAGATGACAA
siRNA #2	CCTCCAGATACACTTTATG

Table S6. Primers used in this study

GAPDH	F	ATGGAGAAGGCTGGGGCTC
	R	AAGTTGTCATGGATGACCTTG
circZFAND6	F	CAGAAGATGTGCAGGGTGTG
	R	GCAGGTGGGCTTATTCTACC
circFAM114A2	F	GTCAAGTATGTAGCAGCTGATGT
	R	TGGAAGGTTTGGTCTCTGGT
circPCCA	F	TGTTATTCGAGGTTGAGCATCC
	R	AGGGGTCCTCAGCATAAACC
circCFL1	F	TGGAGTGACGACTTTGGTTC
	R	CCTCACACTCAGAATCGCCA
circUBE2G1	F	TTCAGGTGAAGGTGGTGTTT
	R	GCGTTCCTCTGGCTTTTCAT
linear UBE2G1	F	GGAACGCTGGCTCCCTATC
	R	GGCTTTTTCTTACACAGCGGG
QKI	F	TGGAAACGAAGGAGAAGCCG
	R	CGCTCGAGGTGGTTGAAGAT
ENO1	F	CCTGCCCTGGTTAGCAAGAA
	R	GGCGTTCGCACCAAACTTAG

Table S7. Sequences of circUBE2G1 RNA and protein

circUBE2G1 RNA:

TGAAGGTGGTGTTTTTAAGGCTCATCTTACTTTCCCAAAAGATTATCCCCTC
CGACCTCCTAAAATGAAATTCATTACAGAAATCTGGCACCCAAATGTTGATA
AAAATGGTGATGTGTGCATTTCTATTCTTCATGAGCCTGGGGAAGATAAGTA
TGGTTATGAAAAGCCAGAGGAACGCTGGCTCCCTATCCACACTGTGGAAA
CCATCATGATTAGTGTCATTTCTATGCTGGCAGACCCTAATGGAGACTCACC
TGCTAATGTTGATGCTGCGAAAGAATGGAGGGAAGATAGAAATGGAGAATT
TAAAAGAAAAGTTGCCCGCTGTGTAAGAAAAAGCCAAGAGACTGCTTTTG
AGTGACATTTATTTAGCAGCTAGTAACTTCACTTATTTCAGG

circUBE2G1-99aa protein:

MKFITEIWHPNVDKNGDVCISILHEPGEDKYGYEKPEERWLPIHTVETIMISVIS
MLADPNGDSPANVDAAKEWREDRNGEFKRKVARCVRKSQETAFE

The start codon (ATG) and stop codon (TGA) are shown in blue letters.

Table S8. Candidate interacting proteins of circUBE2G1-99aa identified by proteomic profiling

Number	Protein names	Gene names
1	Triosephosphate isomerase	TPI1
2	Glyceraldehyde-3-phosphate dehydrogenase	GAPDH
3	Alpha-enolase	ENO1
4	Glucose-6-phosphate isomerase	GPI
5	L-lactate dehydrogenase A chain	LDHA
6	Pyruvate kinase PKM	PKM
7	L-lactate dehydrogenase B chain	LDHB
8	Fructose-bisphosphate aldolase	ALDOA
9	Phosphoenolpyruvate carboxykinase [GTP], mitochondrial	PCK2
10	Filamin-A	FLNA
11	Heat shock cognate 71 kDa protein	HSPA8
12	Tropomyosin alpha-4 chain	TPM4
13	Ubiquitin-60S ribosomal protein L40	UBB
14	Myosin-14	MYH14
15	Unconventional myosin-Ib	MYO1B
16	Heat shock protein HSP 90-beta	HSP90AB1
17	Unconventional myosin-Ic	MYO1C
18	Fascin	FSCN1
19	F-actin-capping protein subunit alpha-1	CAPZA1
20	78 kDa glucose-regulated protein	HSPA5
21	Serpin H1	SERPINH1
22	Gelsolin	GSN
23	Tropomyosin alpha-3 chain	TPM3
24	Elongation factor Tu, mitochondrial	TUFM
25	EF-hand domain-containing protein D1	EFHD1
26	40S ribosomal protein S3	RPS3
27	Putative elongation factor 1-alpha-like 3	EEF1A1
28	Electron transfer flavoprotein subunit alpha, mitochondrial	ETFA
29	ATP synthase subunit beta, mitochondrial	ATP5B
30	Caldesmon	CALD1
31	60S acidic ribosomal protein P0-like	RPLP0
32	Nucleolar RNA helicase 2	DDX21
33	ADP/ATP translocase 2	SLC25A5
34	Heterogeneous nuclear ribonucleoprotein K	HNRNPK
35	KH domain-containing, RNA-binding, signal transduction-associated protein 1	KHDRBS1
36	MICOS complex subunit MIC60	IMMT
37	Ig gamma-2 chain C region	IGHG1
38	Histone H1.2	HIST1H1C
39	60 kDa heat shock protein, mitochondrial	HSPD1
40	60S ribosomal protein L4	RPL4

41	Clathrin heavy chain	CLTC
42	Heterogeneous nuclear ribonucleoprotein C-like 4	HNRNPC
43	Heterogeneous nuclear ribonucleoprotein H	HNRNPH1
44	Tropomodulin-3	TMOD3
45	Heterogeneous nuclear ribonucleoprotein U	HNRNPU
46	Histone H2A	H2AFV
47	Myosin regulatory light chain 12A	MYL12A
48	Tropomyosin alpha-3 chain	TPM3
49	Putative heat shock 70 kDa protein 7	HSPA7
50	40S ribosomal protein S14	RPS14
51	Heat shock protein HSP 90-alpha	HSP90AA1
52	60S ribosomal protein L18a	RPL18A
53	Homeobox protein Hox-A1	HOXA1
54	Heterogeneous nuclear ribonucleoprotein D0	HNRNPD
55	NEDD4-binding protein 2-like 2	N4BP2L2
56	Calcium/calmodulin-dependent protein kinase type II subunit gamma	CAMK2G
57	Coronin	CORO1C
58	Exocyst complex component 3-like protein 4	EXOC3L4
59	Syntaxin-binding protein 3	STXBP3
60	Unconventional myosin-VI	MYO6
61	Filamin A-interacting protein 1-like	FILIP1L
62	Probable ATP-dependent RNA helicase DDX5	DDX5
63	60S ribosomal protein L14	RPL14
64	Myosin light polypeptide 6	MYL6
65	Elongation factor 2	EEF2
66	Axonemal dynein light chain domain-containing protein 1	AXDND1
67	40S ribosomal protein S8	RPS8
68	60S ribosomal protein L8	RPL8
69	Nuclear factor of activated T-cells, cytoplasmic 1	NFATC1
70	Heterogeneous nuclear ribonucleoproteins A2/B1	HNRNPA2B1
71	RNA-binding protein FUS	TAF15
72	R3H domain-containing protein 2	R3HDM2
73	60S ribosomal protein L7a	RPL7A
74	Pyruvate carboxylase, mitochondrial	PC
75	Translocon-associated protein subunit delta	SSR4
76	DNA polymerase zeta catalytic subunit	REV3L
77	Ankyrin repeat and KH domain-containing protein 1	ANKHD1
78	Nucleolin	NCL
79	Protein phosphatase 1 regulatory subunit 37	PPP1R37
80	40S ribosomal protein S13	RPS13
81	Cofilin-2	CFL1
82	Target of rapamycin complex 2 subunit MAPKAP1	MAPKAP1
83	Titin	TTN

84	Focadhesin	FOCAD
85	Protein furry homolog-like	FRYL
86	LETM1 and EF-hand domain-containing protein 1, mitochondrial	LETM1
87	60S ribosomal protein L10a	RPL10A
88	Thioredoxin	TXN
89	Forkhead box protein O1	FOXO1
90	Forkhead box protein O6	FOXO6
91	Plasminogen activator inhibitor 1 RNA-binding protein	SERBP1
92	Secretoglobin family 1D member 2	SCGB1D2
93	Ras GTPase-activating-like protein IQGAP1	IQGAP1
