# Supplementary material for: A novel protein encoded by circUBE2G1 suppresses glycolysis in gastric cancer through binding to ENO1
Source: Cell Death Discov. 2025 Jul 29;11:350. doi: 10.1038/s41420-025-02644-0 (PMC12307642; doi:10.1038/s41420-025-02644-0)
Supplement: Supplementary file 2 — Supplementary figure and figure legend [file 41420_2025_2644_MOESM2_ESM.pdf]

Figure S1. QKI-mediated circUBE2G1 biogenesis encodes a tumor-suppressive peptide that inhibits ENO1-driven glycolysis and metastasis in gastric cancer. **A** Prediction of QKI binding motifs on UBE2G1 pre-mRNA using the RBPsuite database. **B** A schematic illustration of putative binding sites of QKI upstream and downstream of the circUBE2G1 genomic site. **C** Correlation between circUBE2G1 and QKI in 28 GC samples was determined by qRT-PCR with β-actin serving as an internal control. **D** RIP assays were conducted in GES-1 and MKN-28 cells, followed by qRT-PCR to quantify the enrichment of circUBE2G1 in the immunoprecipitated RNA. **E** Western blotting confirmed that the QKI protein interacted with circUBE2G1 in RNA pull-down assays. **F** Changes in circUBE2G1 mRNA and protein levels in different treatment groups after QKI overexpression treatment were detected by RT-qPCR and Western Blot. **G** RT-qPCR analysis showed changes in circUBE2G1 mRNA levels in different GC groups following QKI overexpression treatment. **H** Detection of circUBE2G1-99aa protein expression levels in 60 GC tissues (T) and matched adjacent non-tumor tissues (ANT) by western blotting. **I** Assessment of migration and invasion abilities in GC cells HGC-27 and AGS following transfection with Empty Vector, circUBE2G1-OE, circUBE2G1-ATG mut, or Linear UBE2G1-99aa constructs using Transwell assays (Scale Bar = 200 μm). **J** Effect of circUBE2G1-99aa overexpression on ENO1 mRNA levels detected by qPCR.**K and L** Metabolic profiling of HGC-27 and AGS cells grouped as previously described: assessment of glucose consumption, pyruvate, and lactate production, ATP levels, and bioenergetics via Seahorse XF Analysis. Graph represents mean ± SD; ns, not significant, $*P < 0.05$, $**P < 0.01$, and $***P < 0.001$.

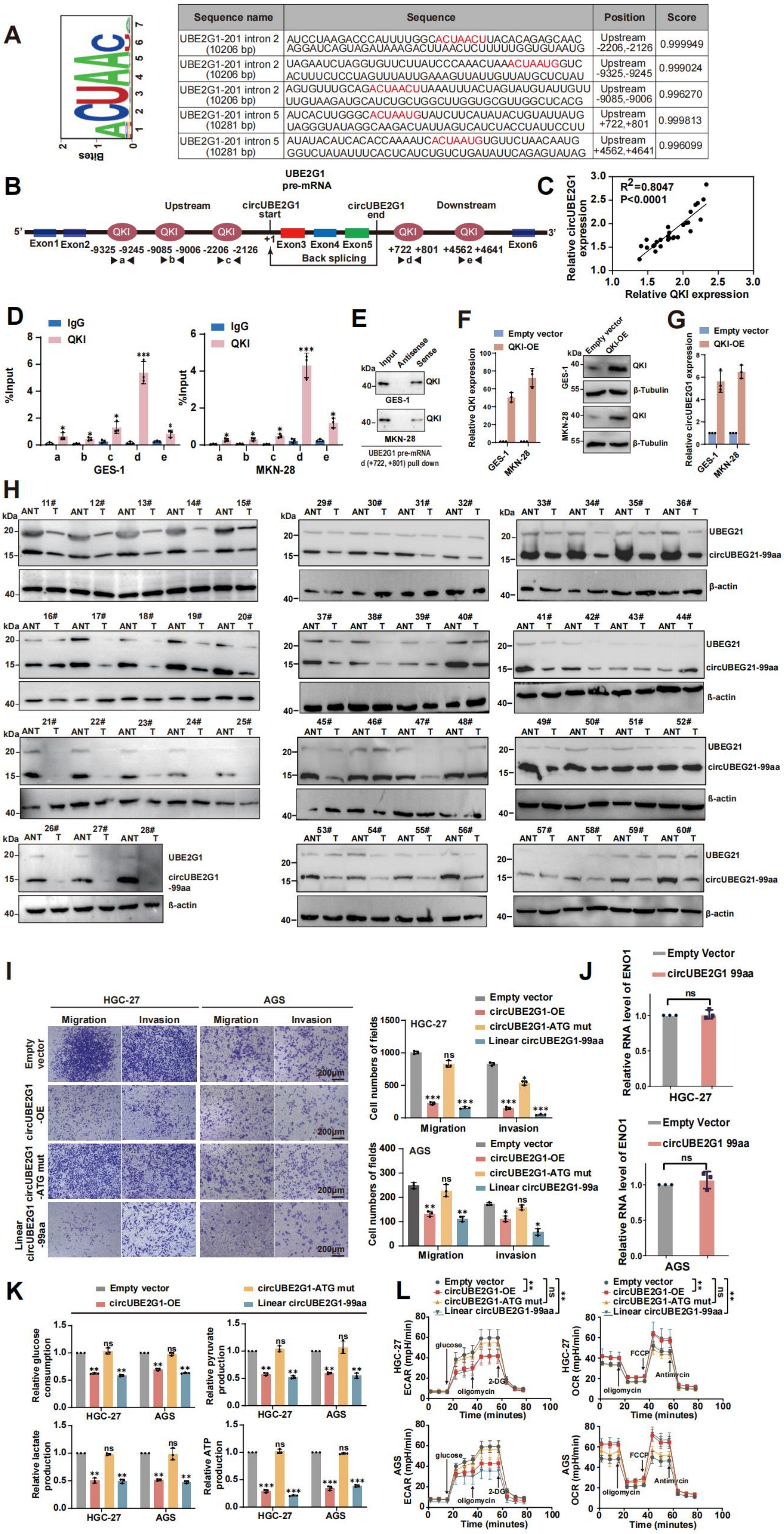

Sequence name	Sequence	Position	Score
UBE2G1-201 intron 2 (10206 bp)	AUCCUAAGACCCAUUUUGGCACUAACUUACACAGAGCAAC AGGAUCAGUAGAUAAAGACUUAACUCUUUUUGGUGUAAUG	Upstream -2206,-2126	0.999949
UBE2G1-201 intron 2 (10206 bp)	UAGAAUCUAGGUGUUCUUAUCCCAAACUAAACUAAUGGUC ACUUUCUCCUAGUUUAUUGAAAGUUAUUGUUAUGCUCUAU	Upstream -9325,-9245	0.999024
UBE2G1-201 intron 2 (10206 bp)	AGUGUUUGCAGACUAACUUAAAUUUACUAGUAUGUAUUGUU UUGUAAGAUGCAUCUGCUGGCUUGGUGCGUUGGCUCACG	Upstream -9085,-9006	0.996270
UBE2G1-201 intron 5 (10281 bp)	AUCACUUGGGCACUAAUGUAUCUUCAUAUACUGUAUUAUG UAGGGUAUAGGCAAGACUAUUAGUCAUCUACCUAUUCCUU	Upstream +722,+801	0.999813
UBE2G1-201 intron 5 (10281 bp)	AUAUACAUCACACCAAAAUCACUAAUGUGUUCUAACAAUG GGUCUAUAUUUCACUCAUCUGUCUGAUAUUCAGAGUAUAG	Upstream +4562,+4641	0.996099
