# Supplementary figures and images for: A novel protein encoded by circUBE2G1 suppresses glycolysis in gastric cancer through binding to ENO1
Source: Cell Death Discov. 2025 Jul 29;11:350. doi: 10.1038/s41420-025-02644-0 (PMC12307642; doi:10.1038/s41420-025-02644-0)

Fig. 1

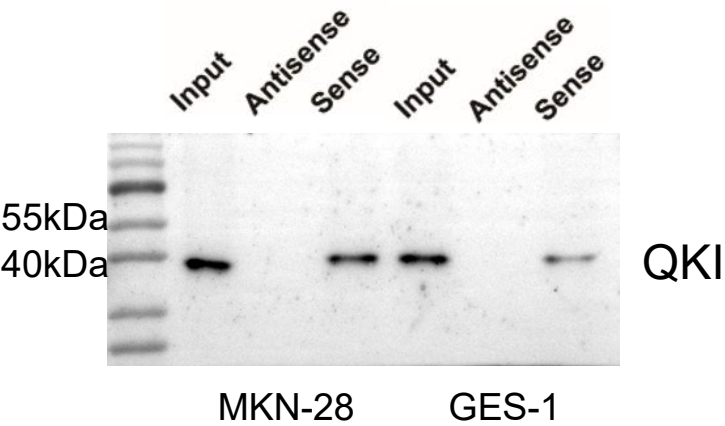

Fig. 1M

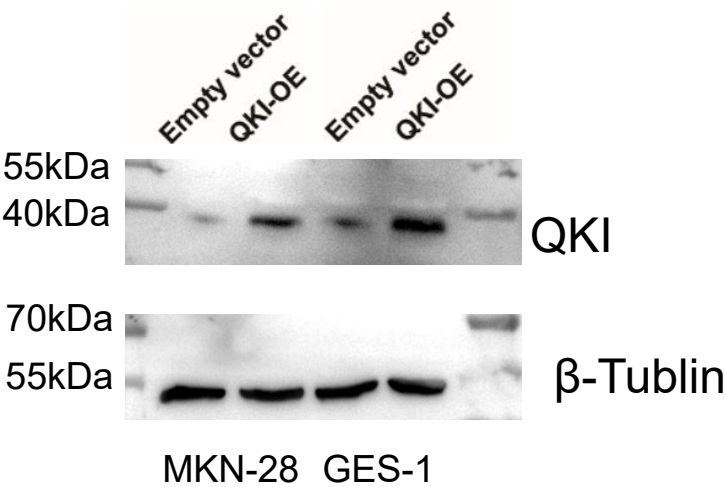

Fig. 1N

Fig. 3

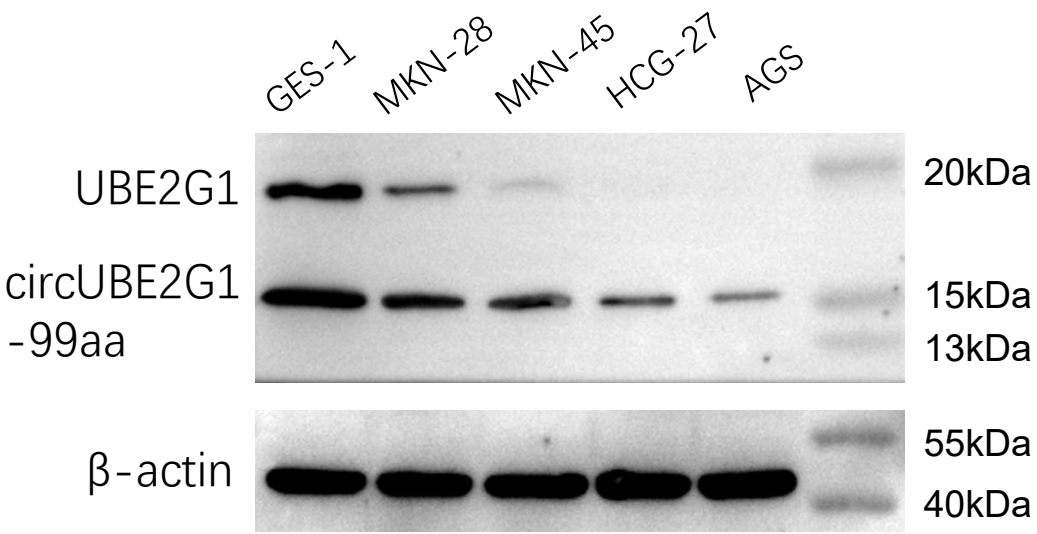

Fig. 3C

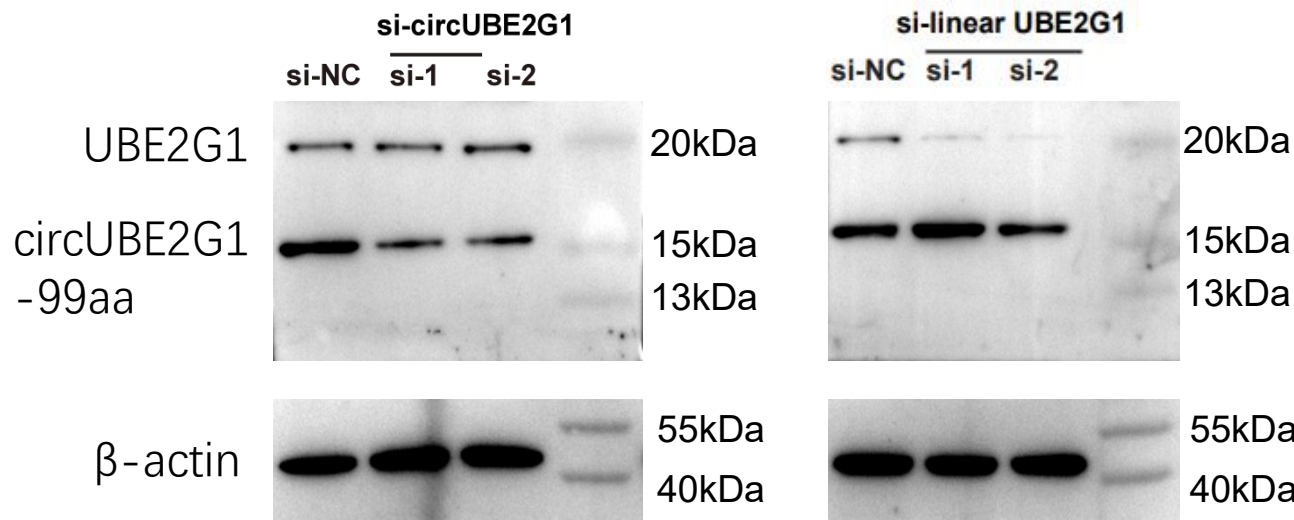

Fig. 3D

Fig. 3H; Fig. s1H

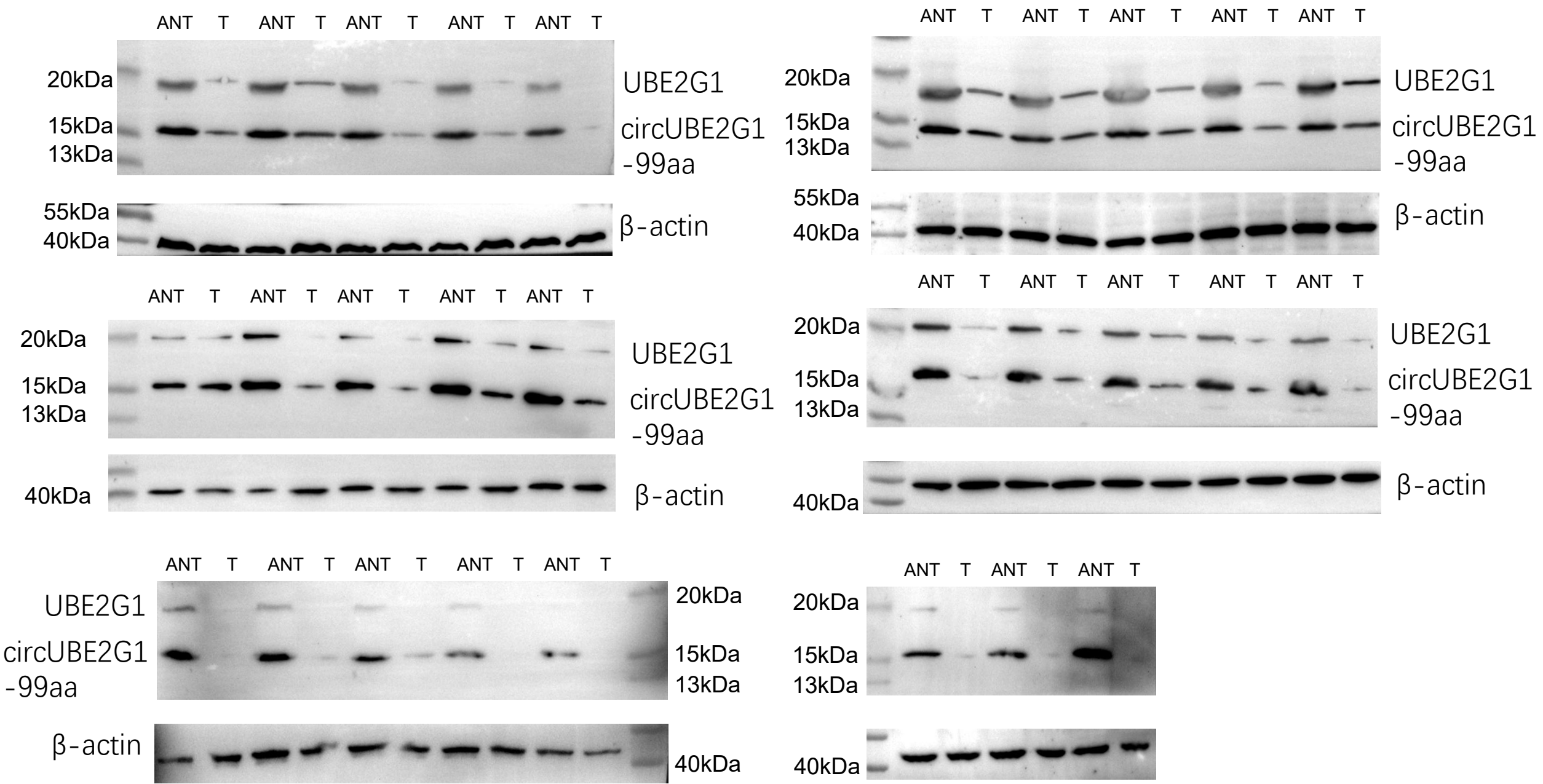

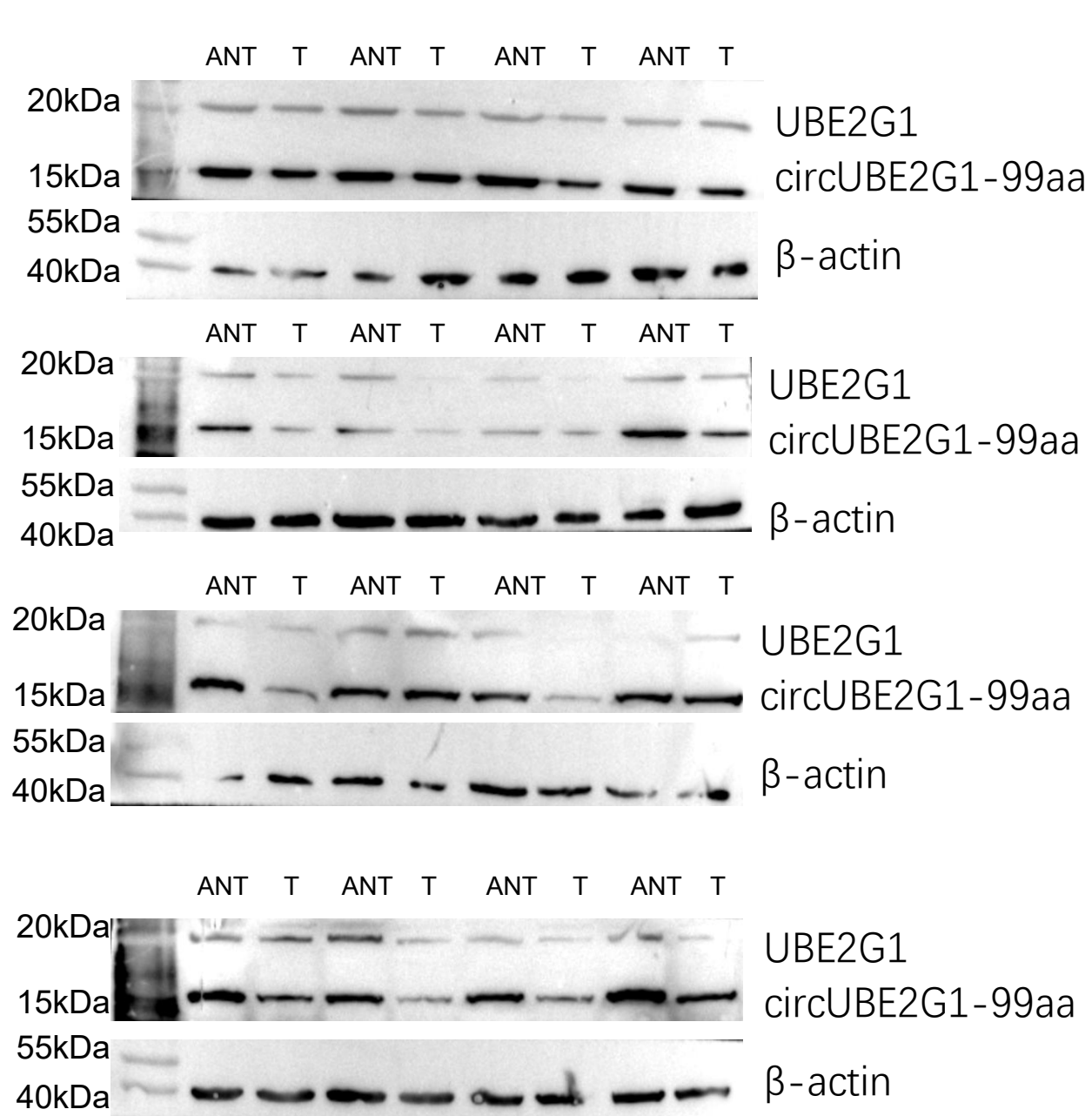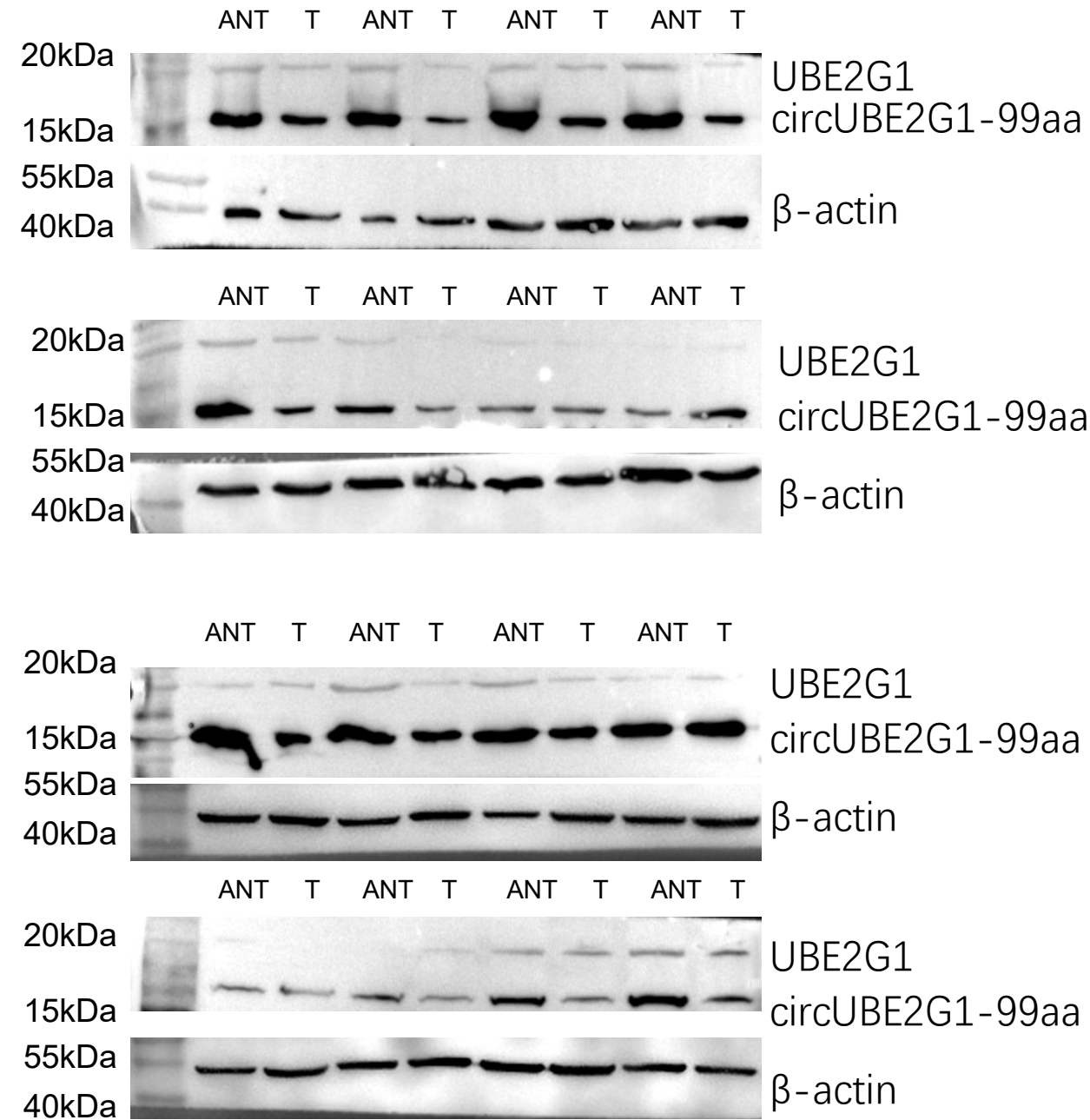

Fig. 4

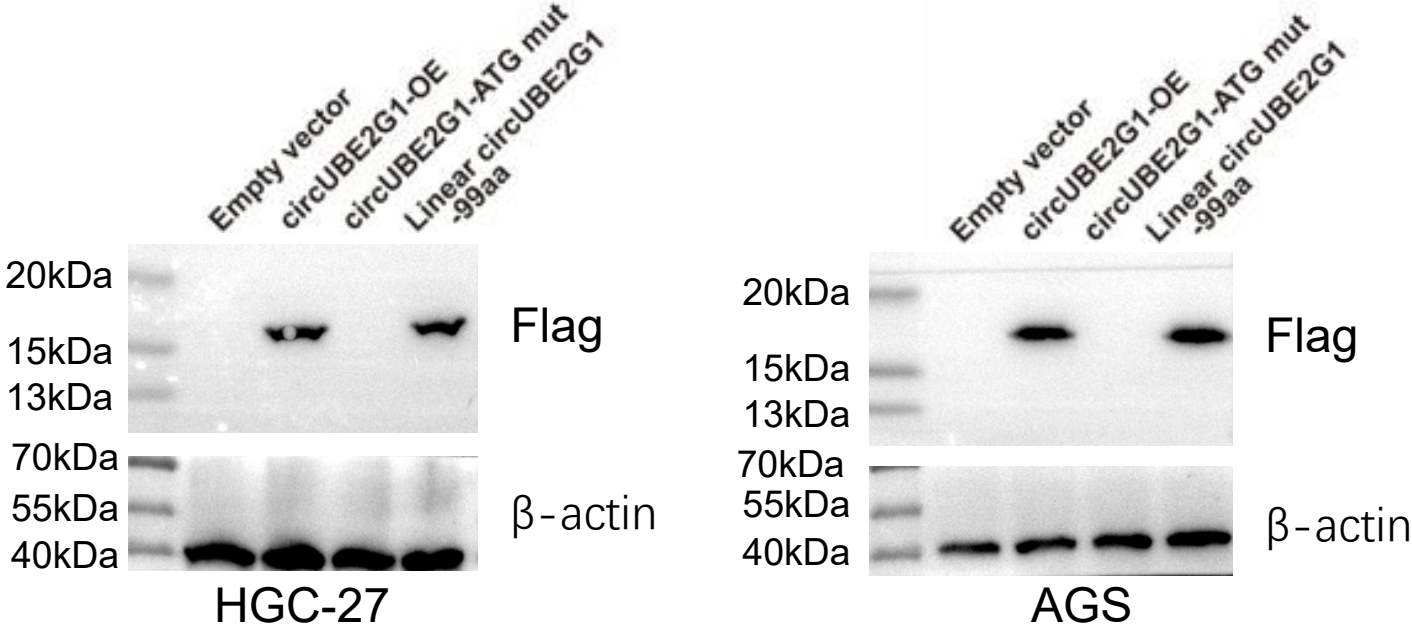

Fig. 4B

Fig. 5

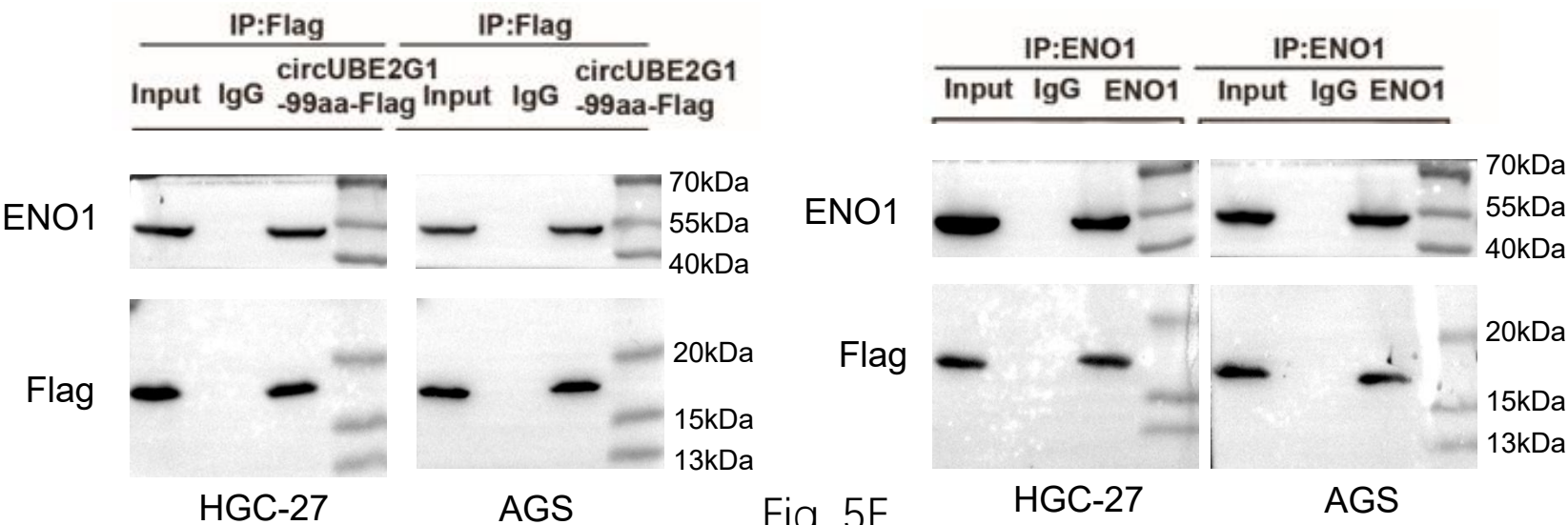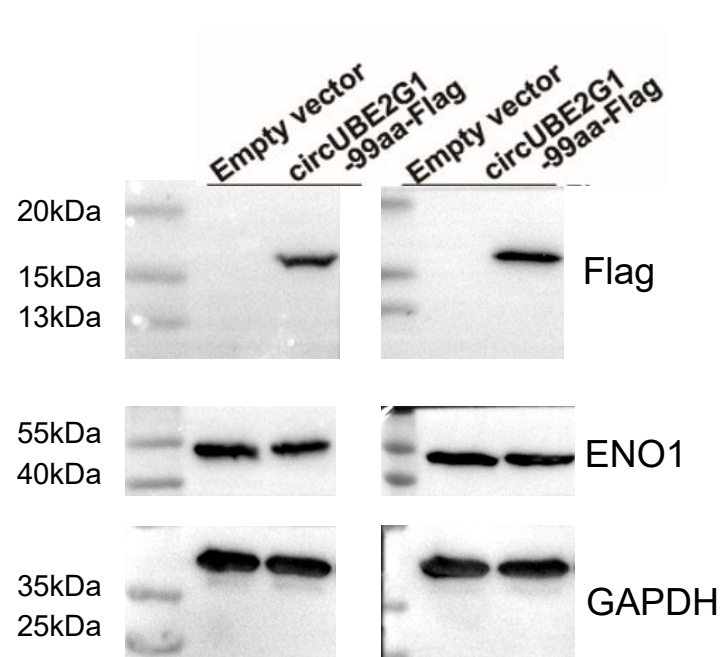

Fig. 5H

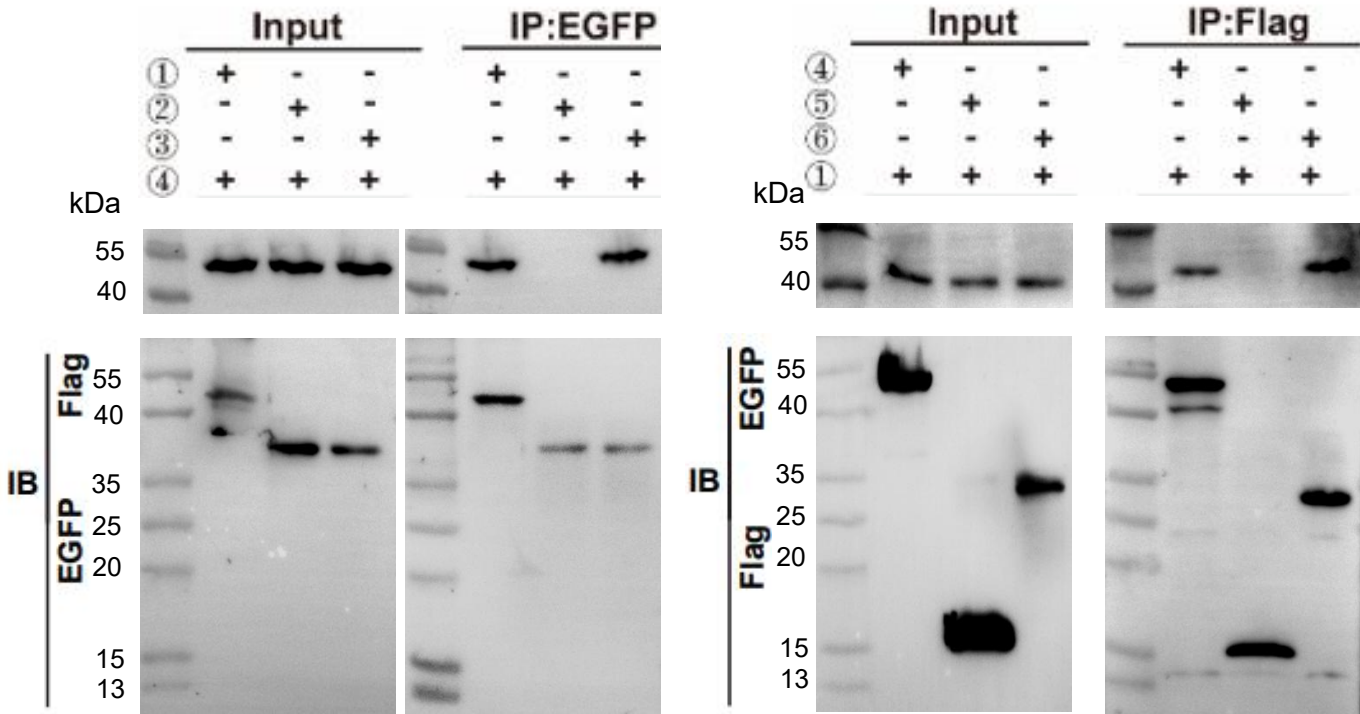

Fig. 5J

Fig. 6

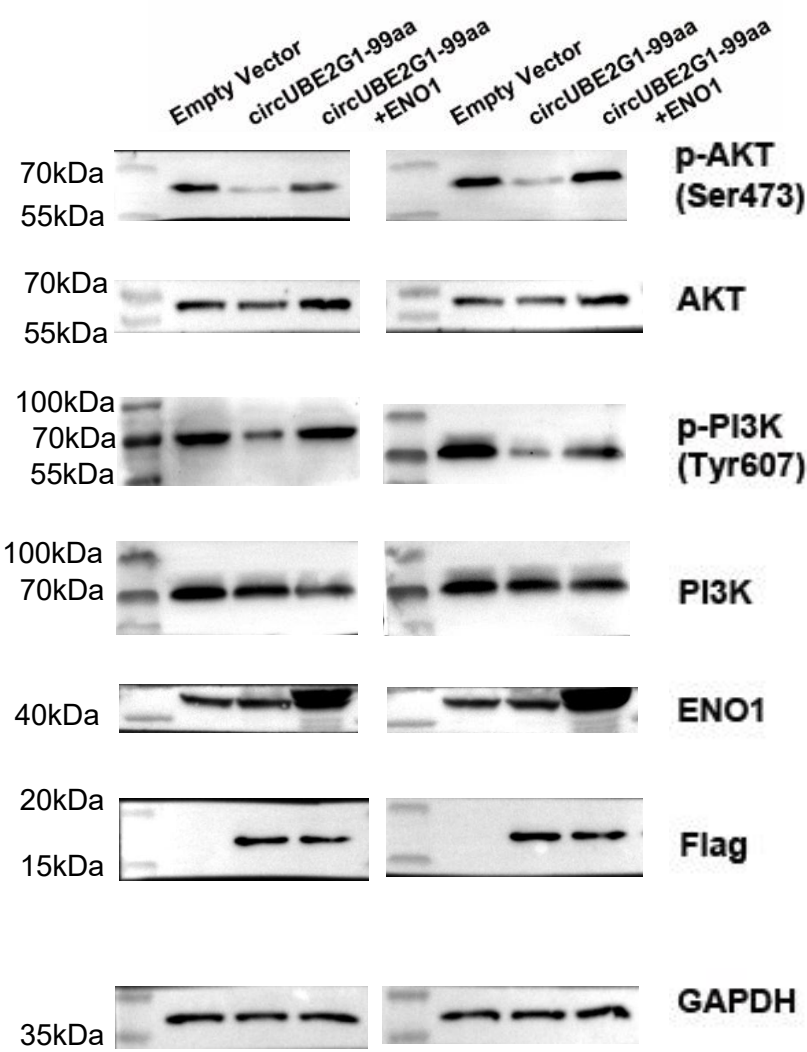

Fig. 6A

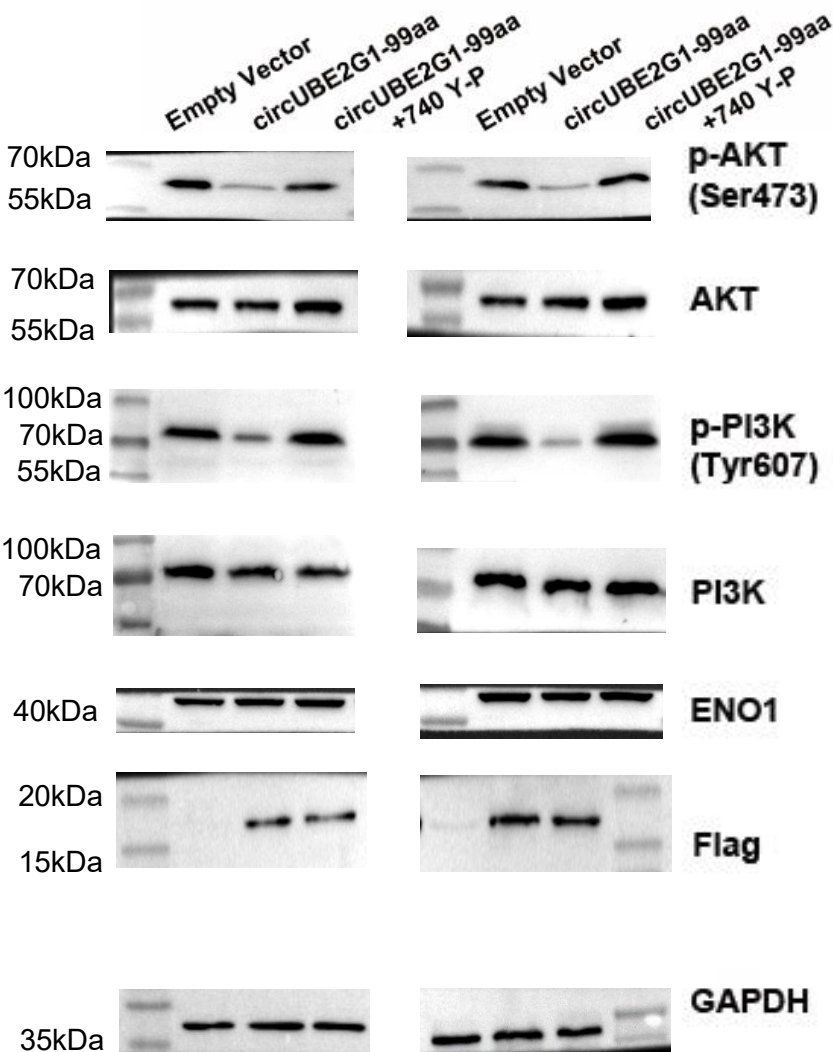

Fig. 6B

Supplement: Supplementary file 3 — WB original drawing+marker [file 41420_2025_2644_MOESM3_ESM.pdf]
